# Changes in Sleep Patterns, Genetic Susceptibility, and Incident Cardiovascular Disease in China

**DOI:** 10.1001/jamanetworkopen.2024.7974

**Published:** 2024-04-23

**Authors:** Tingyue Diao, Kang Liu, Junrui Lyu, Lue Zhou, Yu Yuan, Handong Yang, Tangchun Wu, Xiaomin Zhang

**Affiliations:** 1 Department of Occupational and Environmental Health, Key Laboratory of Environment and Health, Ministry of Education and State Key Laboratory of Environmental Health (Incubating), School of Public Health, Tongji Medical College, Huazhong University of Science and Technology, Wuhan, China; 2 School of Public Health, Guangzhou Medical University, Guangzhou, China; 3 Department of Cardiovascular Diseases, Sinopharm Dongfeng General Hospital, Hubei University of Medicine, Shiyan, China

## Abstract

**Importance:**

The associations of changes in sleep patterns with incident cardiovascular disease (CVD) are not fully elucidated, and whether these associations are modified by genetic susceptibility remains unknown.

**Objectives:**

To investigate the associations of 5-year changes in sleep patterns with incident CVD and whether genetic susceptibility modifies these associations.

**Design, Setting, and Participants:**

This prospective cohort study of the Dongfeng-Tongji cohort was conducted from 2008 to 2018 in China. Eligible participants included those with complete sleep information at baseline survey (2008-2010) and the first follow-up survey (2013); participants who had no CVD or cancer in 2013 were prospectively assessed until 2018. Statistical analysis was performed in November 2023.

**Exposures:**

Five-year changes in sleep patterns (determined by bedtime, sleep duration, sleep quality, and midday napping) between 2008 and 2013, and polygenic risk scores (PRS) for coronary heart disease (CHD) and stroke.

**Main Outcomes and Measures:**

Incident CVD, CHD, and stroke were identified from 2013 to 2018. Cox proportional hazards regression models were applied to estimate hazard ratios (HRs) and 95% CIs.

**Results:**

Among 15 306 individuals (mean [SD] age, 65.8 [7.4] years; 8858 [57.9%] female and 6448 male [42.1%]), 5474 (35.78%) had persistent unfavorable sleep patterns and 3946 (25.8%) had persistent favorable sleep patterns. A total of 3669 incident CVD cases were documented, including 2986 CHD cases and 683 stroke cases, over a mean (SD) follow-up of 4.9 (1.5) years. Compared with those with persistent unfavorable sleep patterns, individuals with persistent favorable sleep patterns over 5 years had lower risks of incident CVD (HR, 0.80; 95% CI, 0.73-0.87), CHD (HR, 0.84; 95% CI, 0.76-0.92), and stroke (HR, 0.66; 95% CI, 0.54-0.82) in the subsequent 5-year period. No significant effect modification by PRS was observed for sleep pattern change and CHD or stroke risk. However, sleep pattern changes and PRS were jointly associated with the CHD and stroke risk in a dose-dependent manner, with the lowest risk being among those with persistent favorable sleep patterns combined with low PRS (HR for CHD, 0.65; 95% CI, 0.52-0.82 and HR for stroke, 0.48; 95% CI, 0.29-0.79).

**Conclusions and Relevance:**

In this cohort study of middle-aged and older Chinese adults, individuals with persistent favorable sleep patterns had a lower CVD risk, even among those with higher genetic risk. These findings highlight the importance of maintaining favorable sleep patterns for CVD prevention.

## Key Points

**Question:**

Are 5-year changes in sleep patterns associated with incident cardiovascular disease (CVD), and, if so, does genetic susceptibility modify these associations?

**Findings:**

In this cohort study of 15 306 individuals in China, those with persistent favorable sleep patterns had a significantly lower risk of new-onset CVD, coronary heart disease, and stroke compared with those with persistent unfavorable sleep patterns. Genetic risk for CVD did not modify these associations; however, sleep pattern changes and genetic risk were jointly associated with the CHD and stroke risk in a dose-dependent manner.

**Meaning:**

These findings suggest that individuals with higher genetic risk may benefit from persistent favorable sleep patterns.

## Teaser

This cohort study explores associations of genetic risk and 5-year changes in sleep patterns with incident cardiovascular disease among retired adults in China.

## Introduction

Cardiovascular disease (CVD) is a major cause of morbidity and mortality worldwide.^1^ In 2019, CVD accounted for approximately one-third of all deaths globally, with more than 40% of deaths in China.^2^ As the CVD burden continues rising in almost all countries, identifying modifiable risk factors for CVD prevention is urgent.

Accumulating evidence^3,4,5,6,7^ has established an association of sleep with cardiovascular health. Several studies^6,8,9,10^ have proposed the development of healthy sleep patterns, assessing sleep as a multidimensional construct, and have shown inverse associations of healthy sleep patterns with the risk of CVD. However, most of these studies have only used a single measurement, which might not adequately reflect the association of overall sleep with CVD because sleep habits may change over time. Only 1 study^10^ of 9309 participants living in Europe showed that maintaining healthy sleep patterns over 2 to 5 years was associated with a lower risk of CVD and coronary heart disease (CHD), but not stroke. However, the study^10^ was conducted among middle-aged people who typically adjusted their sleep patterns around work schedules. Evidence from retired, older people with natural sleep patterns is still lacking.

In addition to lifestyle factors, genetic factors are also associated with CVD.^11,12^ Several studies^13,14,15,16,17^ have explored the joint association of genetic risk and lifestyle with the risk of incident CVD outcomes; these studies showed that individuals adhering to healthy lifestyles had a lower risk of CHD or stroke, even among those at high genetic risk. It is unknown whether maintaining favorable sleep patterns over time is associated with a lower risk of CHD or stroke among individuals with higher genetic susceptibility.

To fill the evidence gap, we collected sleep information at 2 time points approximately 5 years apart, and prospectively explored the long-term outcomes of changes in sleep patterns on the subsequent incidence of CVD outcomes among middle-aged and older Chinese retirees. We further investigated how the 5-year changes in sleep patterns interact and combine with CVD-related genetic variants for the risk of CVD outcomes.

## Methods

### Study Population

This cohort study was approved by the Ethics and Human Participants Committees of Tongji Medical College, Huazhong University of Science and Technology, and Dongfeng General Hospital. This study followed the Strengthening the Reporting of Observational Studies in Epidemiology (STROBE) reporting guideline. The Dongfeng-Tongji cohort is an ongoing prospective cohort study in Shiyan, China.^18^ A total of 27 009 retired workers from the Dongfeng Motor Corporation (DMC) were recruited at the baseline survey between September 2008 and June 2010, and among them, 24 175 participated in the first follow-up survey in 2013. Participants completed standardized questionnaires and medical examinations in both surveys. We excluded participants with diagnosed CHD, stroke, cancer, or severely abnormal electrocardiogram findings before the first follow-up and those with missing sleep information at baseline or the first follow-up survey (eFigure 1 in Supplement 1). Among eligible participants, a subgroup of participants had available genetic data collected. Written informed consent was obtained from all participants.

### Changes in Sleep Patterns

Sleep information was self-reported and collected through standardized questionnaires at baseline from 2008 to 2010 and at the first follow-up in 2013. Details of the assessment are provided in the eMethods in Supplement 1. To assess overall sleep, we created a sleep score based on 4 low-risk sleep factors, details of which were presented in our previous study.^6^ Briefly, low-risk sleep factors were defined as follows: bedtime between 10:01 pm and 12:00 am, sleep duration of 7 to fewer than 8 hours per night, good or fair sleep quality, and midday napping of 60 minutes or less, based on previous knowledge.^6,19,20,21,22,23^ Sleep factors were treated as dichotomous variables (low risk coded as 1 and high risk coded as 0), and sleep score was the sum of all the sleep factors ranging from 0 to 4.

To determine the changes in sleep patterns from baseline to the first follow-up, considering the sample size and associated statistical power, we defined sleep patterns as unfavorable (sleep score ≤2) and favorable (sleep score ≥3) and then divided participants into 4 groups: persistent unfavorable, favorable-unfavorable (ie, transitioning from favorable to unfavorable), unfavorable-favorable (ie, transitioning from unfavorable to favorable), and persistent favorable.

### Polygenic Risk Score

The genotyping arrays used in this study were Illumina Infinium OmniZhongHua-8 chips. Detailed information on the genotyping process was described elsewhere.^24^ The reference panel in genotype imputation was 3931 samples of individuals with East Asian heritage from the 1000 Genomes Project phase III and the SG10K Project. The single-nucleotide variations (SNVs) selected for calculating the polygenic risk scores (PRS) for CHD and stroke were from the recently published and validated CHD PRS^25^ (comprising 540 SNVs) and stroke PRS^26^ (comprising 534 SNVs); of these SNVs, 533 SNVs for CHD (eTable 1 in Supplement 1) and 527 SNVs for stroke (eTable 2 in Supplement 1) reached the criteria of a call rate greater than 95%, minor allele frequency greater than 0.001, *P* value greater than 1 × 10^−6 ^in Hardy-Weinberg equilibrium tests, and a mean *R^2^* imputation quality greater than 0.3 in the Dongfeng-Tongji cohort data. Each SNV was coded as 0, 1, or 2, according to the number of risk alleles it carried. The weighted PRS for CHD and stroke was calculated separately using the following equation:

PRS = (β_1_ × SNV_1_ + β_2_ × SNV_2_ + …β_n−1_ × SNV_n−1_ + β_n_ × SNV_n_) × (N/sum of the β coefficients),

where SNV was coded as 0, 1, or 2 according to the number of risk alleles it carried and the β-coefficient for each SNV was obtained from the previously published CHD and stroke PRS.^25,26^ The CHD PRS and stroke PRS followed normal distribution (eFigure 2 in Supplement 1). The PRS were then classified into low (quintile 1), intermediate (quintile 2–4), or high (quintile 5) genetic risk groups and showed good stratification capability for CHD and stroke (eTable 3 in Supplement 1).

### Outcomes

All participants could be tracked for morbidity and mortality through the DMC health care system and death certificates. The primary outcome in this study was incident CVD, assessed until December 31, 2018. Incident CVD was defined as a composite outcome of incident CHD (*International Statistical Classification of Diseases and Related Health Problems, Tenth Revision* [*ICD-10*] codes I20-I25, and I46) and incident stroke (*ICD-10* codes I60-I61, I63-I64, I69.0-I69.1, and I69.3-I69.4), ascertained by an expert panel of physicians.^27^ Details of the identification and classification of CVD events have been described elsewhere.^22,28^

### Covariates

At baseline and the first follow-up survey, demographic characteristics (age, sex, and education), lifestyle (drinking status, smoking status, and physical activity), and medical history were collected by trained interviewers using standardized questionnaires. Physical examination, including weight, standing height, blood pressure, fasting glucose, and blood lipid levels, were measured by trained physicians (see the eMethods in Supplement 1 for details). The covariates used for adjustment in this study were those collected at the first follow-up survey in 2013.

### Statistical Analysis

The characteristics of participants at follow-up in 2013 were described as mean (SD) for continuous variables and percentages for categorical variables. We calculated the follow-up time for each participant from the date of recruitment at the first follow-up survey to the date of diagnosis of CVD, death, or the censoring date (December 31, 2018), whichever came first. We applied Cox proportional hazard regression models to calculate the hazard ratios (HRs) and 95% CIs. Potential covariates adjusted in the models were age; sex; education level; regular exercise; drinking status; smoking status; body mass index (BMI); hypertension; hyperlipidemia; diabetes; and family history of CVD, CHD, or stroke (in the corresponding analysis). We assessed the assumption of proportional hazards with a test based on Schoenfeld residuals^29^; the nonsignificant results suggested that the models met the assumption. We evaluated HRs and 95% CIs for CVD, CHD, and stroke according to changes in sleep patterns, with a persistent unfavorable sleep pattern being the reference group. We also examined the associations of the changes in sleep patterns with the risk of incident CVD, CHD, and stroke stratified by age (<65 and ≥65 years) and sex (male or female).

In the subgroup of participants with genetic data, we assessed the association of changes in sleep patterns with incident CHD and stroke stratified by each genetic risk group of CHD and stroke, with the potential effect modification tested by including a multiplicative interaction term between the changes in sleep patterns and the genetic risk group. When stratified by PRS, the number of cases among the groups of favorable-unfavorable and unfavorable-favorable sleep patterns was relatively small. Given that both groups represent changed sleep patterns and their associations with CVD risk were similar, we combined them into 1 group in the subsequent analyses to ensure more stable estimates. We further explored the joint associations of changes in sleep patterns and genetic risk of CHD and stroke with incident CHD and stroke, using high genetic risk combined with persistent unfavorable sleep patterns as the reference group. We also performed the linear trend test by treating the joint category as a continuous variable.

We conducted several sensitivity analyses to test the robustness of the results. To reduce the possibility of reverse causation, we repeated the primary analyses after excluding participants with the event of interest observed within the first year of follow-up. To examine whether the results were sensitive to the use of hypnotics, we also reran the primary analyses after excluding participants who reported very poor sleep quality with frequent use of hypnotics. All statistical analyses were performed using R software version 4.2.2 (R Project for Statistical Computing) in November 2023. Statistical significance was defined as a 2-sided *P* value < .05.

## Results

The final cohort included 15 306 participants (mean [SD] age, 65.8 [7.4] years; 6448 male [42.1%] and 8858 female [57.9%]), of which 12 788 had available genetic data. During a mean (SD) follow-up of 4.9 (1.5) years, we documented 3669 incident CVD cases, including 2986 CHD cases and 683 stroke cases. Table 1 shows the participant characteristics in 2013 across 5-year changes in sleep patterns. Of the 15 306 participants, 5474 (35.8%) had persistent unfavorable sleep patterns and 3946 (25.8%) had persistent favorable sleep patterns. Participants with persistent favorable sleep patterns were more likely to be younger, female, and better educated.

**Table 1.** Characteristics of the Study Participants at the First Follow-Up Survey in 2013 According to Changes in Sleep Patterns

Characteristics	Participants, No. (%) (N = 15 306)			
	Persistent unfavorable (n = 5474)	Favorable-unfavorable (n = 3119)	Unfavorable-favorable (n = 2767)	Persistent favorable (n = 3946)
Age, mean (SD), y	67.1 (7.5)	65.9 (7.4)	65.0 (7.3)	64.6 (7.1)
Sex				
Male	2580 (47.1)	1287 (41.3)	1113 (40.2)	1468 (37.2)
Female	2894 (52.9)	1832 (58.7)	1654 (59.8)	2478 (62.8)
Education level				
Primary school or below	2018 (36.9)	869 (27.9)	694 (25.1)	687 (17.4)
Middle school	2027 (37.0)	1156 (37.1)	1048 (37.9)	1421 (36.0)
High school or beyond	1388 (25.4)	1069 (34.3)	1005 (36.3)	1818 (46.1)
Current smoking	864 (15.8)	475 (15.2)	406 (14.7)	569 (14.4)
Current drinking	1379 (25.2)	724 (23.2)	652 (23.6)	938 (23.8)
Regular exercise	4390 (80.2)	2481 (79.5)	2204 (79.7)	3195 (81.0)
Body mass index, mean (SD)^a^	24.1 (3.2)	24.1 (3.2)	24.2 (3.2)	24.1 (3.1)
Hypertension	3287 (60.0)	1913 (61.3)	1695 (61.3)	2434 (61.7)
Diabetes	851 (15.5)	441 (14.1)	466 (16.8)	613 (15.5)
Hyperlipidemia	2261 (41.3)	1276 (40.9)	1157 (41.8)	1671 (42.3)
Family history of cardiovascular disease	682 (12.5)	503 (16.1)	458 (16.6)	821 (20.8)

^a^ Body mass index was calculated as weight in kilograms divided by height in meters squared.

**Figure 1.**
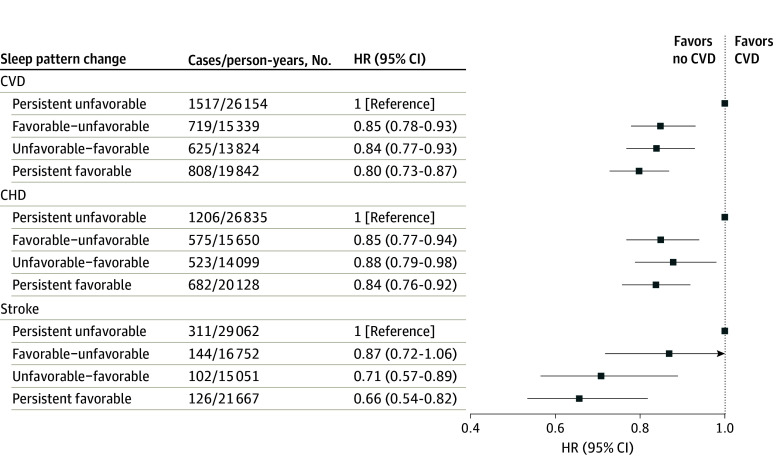
Associations of Changes in Sleep Patterns With Risk of Incident Cardiovascular Disease (CVD) The Cox regression models were adjusted for age, sex, education level, smoking status, drinking status, regular exercise, body mass index, hypertension, diabetes, hyperlipidemia, and family history of CVD, coronary heart disease (CHD) or stroke (in the corresponding analysis). HR, indicates hazard ratio.

Figure 1 illustrates the associations of the 5-year changes in sleep patterns with the subsequent risk of incident CVD, CHD, and stroke. Compared with persistent unfavorable sleep patterns, the risk of CVD was lower for those with favorable-unfavorable sleep patterns (adjusted HR [aHR], 0.85; 95% CI, 0.78-0.93), those with unfavorable-favorable sleep patterns (aHR, 0.84; 95% CI, 0.77-0.93), and those with persistent favorable sleep patterns (aHR, 0.80; 95% CI, 0.73-0.87). Changes in sleep patterns were also associated with lower risk of CHD and stroke; participants with persistent favorable sleep patterns had a 16% lower risk of CHD (HR, 0.84; 95% CI, 0.76-0.92) and 34% lower risk of stroke (HR, 0.66; 95% CI, 0.54-0.82). The associations of changes in sleep patterns with CVD, CHD, and stroke were consistent in subgroup analyses stratified by age and sex (eTable 4 in Supplement 1).

Table 2 presents the PRS-stratified analyses of the association of 5-year changes in sleep patterns with incident CHD and stroke. Sleep patterns were not associated with risk of incident CHD among those with low or high genetic risk (*P* for interaction = .36). Persistent favorable sleep patterns were associated with a 15% lower risk of CHD in the intermediate genetic risk subgroup (aHR, 0.85; 95% CI, 0.74-0.98). For stroke, there was also no evidence of stroke PRS being a modifier (*P* for interaction = .23). Among individuals with persistent favorable sleep patterns, those at low genetic risk had a lower but not statistically significant risk of incident stroke (aHR, 0.77; 95% CI, 0.44-1.35), whereas there was a 36% lower risk of stroke for those at intermediate genetic risk (aHR, 0.64; 95% CI, 0.47-0.86) and 45% lower risk of stroke for those at high genetic risk (aHR, 0.55; 95% CI, 0.33-0.93).

**Table 2.** Associations of Changes in Sleep Patterns With Coronary Heart Disease and Stroke, Stratified by the Genetic Risk Group

Incidence	Sleep pattern change			*P* for interaction
	Persistent unfavorable	Changed	Persistent favorable	
**Coronary heart disease**				
Low genetic risk				.36
Cases/person-years	192/4475	163/4994	109/3515	
HR (95% CI)^a^	1 [Reference]	0.81 (0.65-1.00)	0.82 (0.64-1.05)	
Intermediate genetic risk				
Cases/person-years	582/13 531	566/14 723	343/10 369	
HR (95% CI)^a^	1 [Reference]	0.95 (0.84-1.06)	0.85 (0.74-0.98)	
High genetic risk				
Cases/person-years	224/4279	208/4984	144/3285	
HR (95% CI)^a^	1 [Reference]	0.85 (0.70-1.02)	0.96 (0.77-1.19)	
**Stroke**				
Low genetic risk				.23
Cases/person-years	37/4699	37/5241	20/4001	
HR (95% CI)^a^	1 [Reference]	0.96 (0.60-1.51)	0.77 (0.44-1.35)	
Intermediate genetic risk				
Cases/person-years	153/14 705	120/16 024	60/10 769	
HR (95% CI)^a^	1 [Reference]	0.80 (0.63-1.01)	0.64 (0.47-0.86)	
High genetic risk				
Cases/person-years	60/4772	38/5268	20/3764	
HR (95% CI)^a^	1 [Reference]	0.64 (0.42-0.97)	0.55 (0.33-0.93)	

Abbreviation: HR, hazard ratio.

^a^ The Cox regression models were adjusted for age, sex, education level, smoking status, drinking status, regular exercise, body mass index, hypertension, diabetes, hyperlipidemia, and family history of coronary heart disease or stroke (in the corresponding analysis).

**Figure 2.**
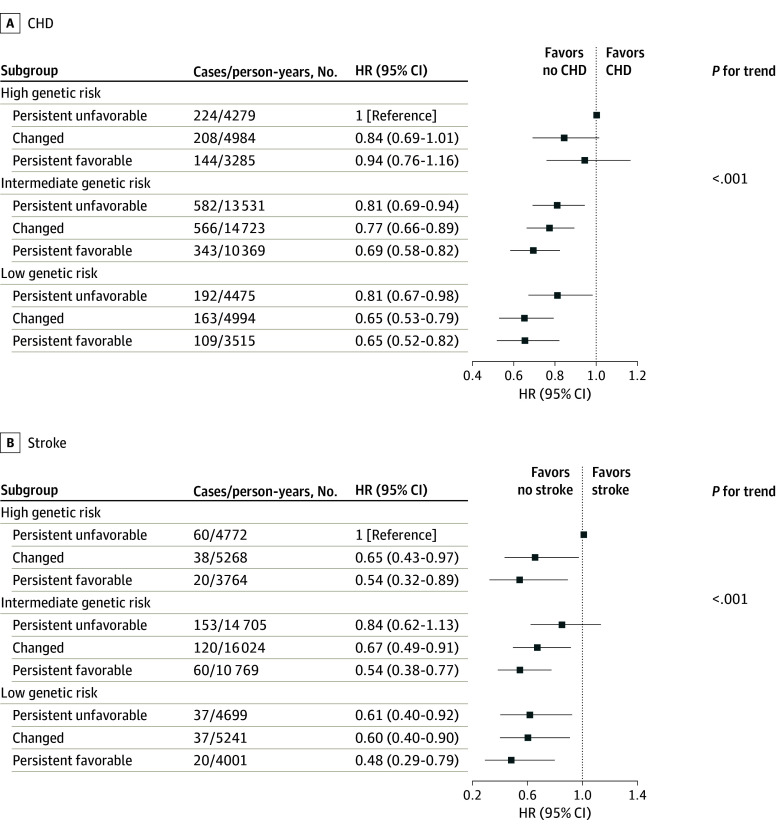
Joint Associations of Changes in Sleep Patterns and Genetic Risk With Coronary Heart Disease (CHD) and Stroke The Cox regression models were adjusted for age, sex, education level, smoking status, drinking status, regular exercise, body mass index, hypertension, diabetes, hyperlipidemia, and family history of CHD or stroke (in the corresponding analysis). HR indicates hazard ratio.

Figure 2 shows the joint associations of 5-year changes in sleep patterns and genetic risk of CHD and stroke with the subsequent risk of incident CHD and stroke. We observed a generally monotonic association of decreasing PRS and changes in sleep patterns (from persistent unfavorable to persistent favorable) with a lower risk of incident CHD (*P* for linear trend < .001) and stroke (*P* for linear trend < .001). Compared with those with persistent unfavorable sleep patterns and high genetic risk, participants with persistent favorable sleep patterns and low genetic risk had a 35% risk of CHD (HR, 0.65; 95% CI, 0.52-0.82) and a 52% lower risk of stroke (HR, 0.48; 95% CI, 0.29-0.79).

Sensitivity analyses restricted to participants with follow-up over 1 year yielded similar results (eTable 5 in Supplement 1). Removing participants who reported very poor sleep quality with frequent use of hypnotics did not change the results (eTable 6 in Supplement 1). We calculated correlations between individual sleep factors and found correlations between bedtime and sleep duration (eFigure 3 in Supplement 1).

## Discussion

In this prospective cohort study, individuals with persistent favorable sleep patterns over 5 years had the lowest risk of incident CVD, CHD, and stroke during the subsequent 5 years. There was no evidence of effect modification by genetic susceptibility to CHD or stroke. Joint subgroups of changes in sleep patterns and genetic risk were inversely associated with the risk of CHD and stroke, with the lowest risk found in individuals at low genetic risk who maintained favorable sleep patterns over 5 years.

Little is known about the associations of longitudinal changes in sleep patterns with subsequent risk of CVD outcomes. We found that retired Chinese adults with persistent favorable sleep patterns over 5 years had the lowest risk of developing CVD, CHD, and stroke. The only previous study^10^ of longitudinal changes in sleep patterns and CVD among 9309 European individuals obtained comparable results to ours, except for stroke risk. This discrepancy may be mainly due to the limited incident strokes (72 cases) in their study. Additionally, we found that even individuals with favorable sleep patterns at a single time point during the 5 years had a lower risk of subsequent CVD outcomes than those with persistent unfavorable sleep patterns. This finding provides further evidence for the beneficial outcomes of favorable sleep patterns.

We did not find evidence of PRS as an effect modifier. In the PRS-stratified analyses, even among participants with a higher genetic predisposition, adherence to favorable sleep patterns was associated with a reduced risk of CHD or stroke. When assessing changes in sleep patterns and genetic risk jointly, as expected, we found the lowest risk of incident CHD and stroke in individuals with low genetic risk for CHD and stroke combined with persistent favorable sleep patterns over 5 years. Similar to our findings, a previous study^8^ utilizing single-measurement sleep data from the UK Biobank suggested that individuals with poor sleep patterns and high genetic risk had the highest risk of CHD and stroke, with low genetic risk combined with healthy sleep patterns as the reference. However, their results showed no difference in risk of stroke when comparing individuals with poor sleep and high genetic risk with those with healthy sleep patterns and low genetic risk.^8^ By using repeated sleep measurements, we found that individuals with low genetic risk and persistent favorable sleep patterns over 5 years had a significantly lower risk of stroke than those with high genetic risk and persistent unfavorable sleep patterns. Taken together, our findings suggest that maintaining favorable sleep patterns over time is associated with benefits in CVD prevention, even in individuals with higher genetic risk. It should be noted that these results need to be interpreted with caution, given the observational nature of this study and the possibility of chance findings due to multiple testing. Further studies, including powered, randomized clinical trials on sleep intervention, are warranted to substantiate our findings. If confirmed, this finding could have important public health implications, especially for those with high genetic risk. Because sleep patterns are modifiable and genetic risk may be immutable, it is crucial to implement appropriate strategies to promote long-term sleep health.

### Limitations

Several potential limitations warrant comment. First, we calculated sleep duration based on 2 questions about bedtime and wake-up time, which may not have allowed us to distinguish sleep time from time spent in bed and might, therefore, have overestimated actual sleep duration. However, these questions are a widely used measure of sleep duration in large epidemiological cohort studies.^7,30,31^ Second, we did not collect information on sleep disorders (such as sleep apnea) and depression, which might have confounded the associations. Third, our participants were middle-aged and older Chinese retirees, which may limit the generalizability of the results. Fourth, we cannot draw any conclusions about causality because this is an observational study.

## Conclusions

In this cohort study, persistent favorable sleep patterns over 5 years were associated with a significantly lower risk of incident CVD outcomes during the subsequent 5 years. For individuals with higher genetic risk, those with persistent favorable sleep patterns had a lower risk of CHD and stroke. These findings highlight the importance of maintaining favorable sleep patterns over time.

## Notes

Supplement 1. **eFigure 1.** Flow Chart of Participants Ultimately Included in This Study
eMethods.
**eTable 1.** Genetic Variants Included in the CHD PRS **eTable 2.** Genetic Variants Included in the Stroke PRS **eFigure 2.** Distributions of CHD PRS and Stroke PRS **eTable 3.** Associations of CHD PRS and Stroke PRS With Risk of Incident CHD and Stroke **eTable 4.** Associations of Changes in Sleep Patterns With Risk of Incident CVD According to Age and Sex **eTable 5.** Associations of Changes in Sleep Patterns With Risk of Incident CVD After Excluding Events Occurred Within the First Year of Follow-Up **eTable 6.** Associations of Changes in Sleep Patterns With Risk of Incident CVD After Excluding Participants Reporting Very Poor Sleep Quality With Frequent Use of Hypnotics **eFigure 3.** Spearman Correlation Between Individual Sleep Factors
eReferences

Supplement 2.
Data Sharing Statement
