# Supplementary material for: Changes in Sleep Patterns, Genetic Susceptibility, and Incident Cardiovascular Disease in China
Source: JAMA Netw Open. 2024 Apr 23;7(4):e247974. doi: 10.1001/jamanetworkopen.2024.7974 (PMC11040405; doi:10.1001/jamanetworkopen.2024.7974)
Supplement: Supplement 1. — eFigure 1. Flow Chart of Participants Ultimately Included in This Study eMethods. eTable 1. Genetic Variants Included in the CHD PRS eTable 2. Genetic Variants Included in the Stroke PRS eFigure 2. Distributions of CHD PRS and Stroke PRS eTable 3. Associations of CHD PRS and Stroke PRS With Risk of Incident CHD and Stroke eTable 4. Associations of Changes in Sleep Patterns With Risk of Incident CVD According to Age and Sex eTable 5. Associations of Changes in Sleep Patterns With Risk of Incident CVD After Excluding Events Occurred Within the First Year of Follow-Up eTable 6. Associations of Changes in Sleep Patterns With Risk of Incident CVD After Excluding Participants Reporting Very Poor Sleep Quality With Frequent Use of Hypnotics eFigure 3. Spearman Correlation Between Individual Sleep Factors eReferences [file jamanetwopen-e247974-s001.pdf]

Supplemental Online Content

Diao T, Liu K, Lyu J, et al. Changes in sleep patterns, genetic susceptibility, and incident cardiovascular disease in China. JAMA Netw Open. 2024;7(4):e247974. doi:10.1001/jamanetworkopen.2024.7974

eFigure 1. Flow Chart of Participants Ultimately Included in This Study
eMethods.
eTable 1. Genetic Variants Included in the CHD PRS
eTable 2. Genetic Variants Included in the Stroke PRS
eFigure 2. Distributions of CHD PRS and Stroke PRS
eTable 3. Associations of CHD PRS and Stroke PRS With Risk of Incident CHD and Stroke
eTable 4. Associations of Changes in Sleep Patterns With Risk of Incident CVD According to Age and Sex
eTable 5. Associations of Changes in Sleep Patterns With Risk of Incident CVD After Excluding Events Occurred Within the First Year of Follow-Up
eTable 6. Associations of Changes in Sleep Patterns With Risk of Incident CVD After Excluding Participants Reporting Very Poor Sleep Quality With Frequent Use of Hypnotics
eFigure 3. Spearman Correlation Between Individual Sleep Factors
eReferences

This supplemental material has been provided by the authors to give readers additional information about their work.

© 2024 Diao T et al. *JAMA Network Open.*

eFigure 1. Flow Chart of Participants Ultimately Included in This Study

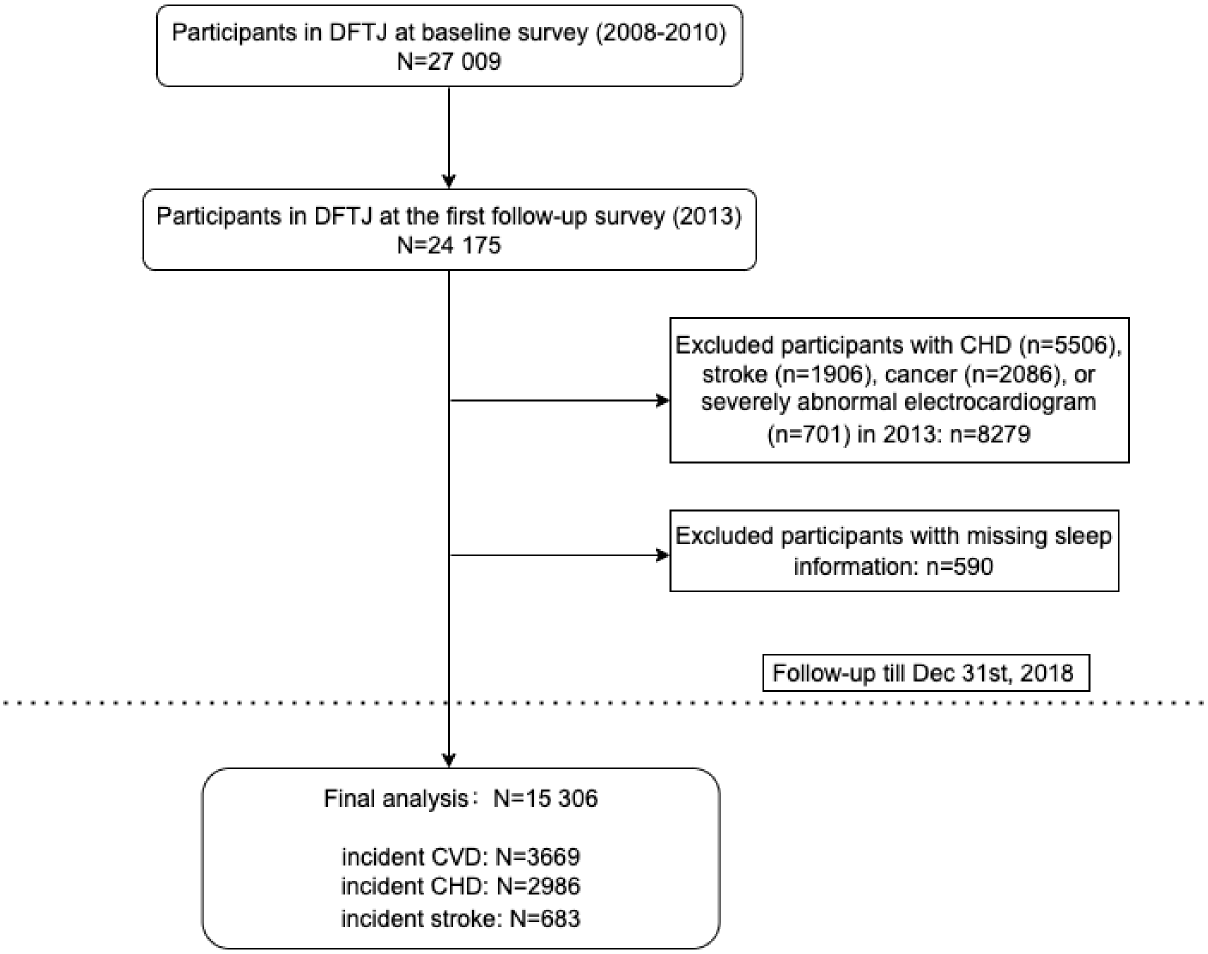

Abbreviations: DFTJ, Dongfeng-Tongji; CHD, coronary heart disease; CVD, cardiovascular disease.

© 2024 Diao T et al. *JAMA Network Open.*

eMethods

Assessment of sleep factors

Bedtime was assessed by the question 'What time did you usually go to sleep at night over the past 6 months?' and then divided into 5 groups: ≤ 9:00 p.m., 9:01 p.m.-10:00 p.m., 10:01 p.m.-11:00 p.m., 11:01 p.m.-12:00 a.m., and > 12:00 a.m. A similar question was used to obtain the wake-up time. Sleep duration was the time interval between bedtime and wake-up time. Sleep quality was evaluated by the question 'How was your sleep quality at night in the past 6 months?' and categorized into good, fair, poor, and very poor with frequent use of hypnotics (combined into poor). Midday napping was assessed by self-reported average midday napping duration. The above-mentioned questions have also been applied to assess bedtime, sleep duration, sleep quality, and napping in other large population-based studies.[1-3]

Assessment of covariates

Education level was coded as primary school or below, middle school, and high school or beyond. Current smokers were defined as those smoking at least 1 cigarette/day over the past 6 months. Current drinkers were defined as those drinking at least once per week over the past 6 months. Physical activity was defined as maintaining a minimum of 7.5 weekly metabolic equivalent of task-hours (MET-hours/week), equivalent to the 2010 physical activity recommendations from WHO. Details of the calculation of MET-hours/week were reported in a previous study from our lab.[4] Body mass index (BMI) was calculated as weight in kilograms divided by height squared in meters. Hypertension was defined as measured blood pressure ≥ 140/90 mm Hg, taking medications for hypertension, or self-reported physician-diagnosed hypertension. Diabetes was defined as fasting glucose ≥ 7.0 mmol/L, taking medications for diabetes, or self-reported physician-diagnosed diabetes. Hyperlipidemia was defined as total cholesterol ≥ 6.22 mmol/L, triglycerides > 2.26 mmol/L, high-density lipoprotein cholesterol < 1.04 mmol/L, low-density lipoprotein cholesterol ≥ 4.14 mmol/L, taking lipid-lowering medications, or self-reported physician diagnosis.

© 2024 Diao T et al. *JAMA Network Open.*

eTable 1. Genetic Variants Included in the CHD PRS

Genetic variants	Chr:Pos hg19	Genes	Effect allele	Other allele	Traits	Weight
rs36096196	1:2252205	*MORN1*	T	C	CAD	0.0392
rs17035646	1:10796547	*CASZ1*	A	G	BP	-0.0113
rs880315	1:10796866	*CASZ1*	T	C	BP;stroke	-0.0185
rs4846049	1:11850365	*MTHFR*	T	G	BP	-0.002
rs12027135	1:25775733	*TMEM57*	T	A	TC;LDL	0.0046
rs10890238	1:38445654	*SF3A3*	T	A	CAD	0.0506
rs2296172	1:39835817	*MACF1*	G	A	HDL	0.007
rs11205760	1:51174330	*FAF1*	C	T	T2D	-0.0268
rs11206510	1:55496039	*PCSK9*	C	T	CAD	-0.0417
rs7525649	1:55499156	*PCSK9*	C	T	LDL;TC	-0.0481
rs151193009	1:55509585	*PCSK9*	T	C	LDL;TC	-0.44
rs9970807	1:56965664	*PLPP3*	T	C	CAD	-0.0972
rs10889353	1:63118196	*DOCK7*	C	A	TC;TG	-0.0083
rs2815752	1:72812440	*RPL31P12*	G	A	BMI	-0.0143
rs1514175	1:74991644	*FPGT-TNNI3K*	G	A	BMI	-0.0195
rs17381664	1:78048331	*ZZZ3*	C	T	BMI	-0.1693
rs117711462	1:93159927	*EVI5*	A	G	LDL;TC	0.0311
rs1555543	1:96944797	*EEF1A1P11*	A	C	BMI	-0.0132
rs7528419	1:109817192	*CELSR2*	G	A	CAD	0.0026
rs12740374	1:109817590	*CELSR2*	T	G	LDL;TC	-0.1091
rs12037987	1:113042822	*WNT2B*	C	T	stroke	0.0219
rs6537746	1:113163529	*CAPZA1*	G	A	BP	0
rs10745332	1:113189053	*CAPZA1*	G	A	BP	-0.0313
rs17030613	1:113190807	*CAPZA1*	C	A	BP	0.0274
rs984222	1:119503843	*TBX15*	C	G	WHR	0.0013
rs10923931	1:120517959	*NOTCH2*	T	G	T2D	0.0053
rs11810571	1:151762308	*TDRKH*	C	G	CAD	-0.0234
rs67156297	1:154336716	*RP11-350G8.4*	A	G	T2D	0.0097
rs4845625	1:154422067	*IL6R*	T	C	CAD	0.0412
rs1052053	1:156202173	*PMF1-SEMA4A*	G	A	stroke	-0.0045
rs2758607	1:156202759	*PMF1-BGLAP*	A	G	stroke	-4.00E-04
rs1892094	1:169094459	*ATP1B1*	T	C	CAD	-0.042
rs2213732	1:172333595	*DNM3*	G	A	WHR	0.0203
rs574367	1:177873210	*SEC16B*	T	G	BMI	0.068
rs1689800	1:182168885	*LINC01344*	G	A	HDL	0
rs6700559	1:200646073	*RP11-92G12.3*	C	T	CAD	0.0254

© 2024 Diao T et al. *JAMA Network Open.*

rs2820315	1:201872264	*LMOD1*	T	C	CAD	0.0017
rs2819348	1:201884952	*LMOD1*	C	T	CAD	0.0646
rs60154123	1:210468999	*RP4-667H12.4*	T	C	CAD	3.00E-04
rs2075423	1:214154719	*PROX1*	T	G	T2D	-0.0053
rs340874	1:214159256	*PROX1*	C	T	T2D	0.0049
rs2820443	1:219753509	*RP11-95P13.2*	C	T	WHR	0.0096
rs2642442	1:220973563	*NA*	C	T	TC;LDL	-0.0026
rs17465637	1:222823529	*MIA3*	A	C	CAD	-0.0877
rs2144300	1:230294916	*GALNT2*	T	C	TG	-0.0117
rs699	1:230845794	*AGT*	A	G	CAD	-0.0334
rs2783963	1:243501583	*SDCCAG8*	A	G	BMI	-0.0029
rs12463617	2:629244	*TMEM18*	A	C	BMI	-0.0838
rs1344653	2:19730845	*LINC01808*	G	A	BP	0.0137
rs16986953	2:19942473	*AC019055.1*	A	G	CAD	0.0708
rs2123536	2:19945577	*AC019055.1*	T	C	CAD	0.0041
rs13306194	2:21252534	*APOB*	A	G	LDL;TC;TG	-0.0888
rs1367117	2:21263900	*APOB*	A	G	TC;LDL	0.0078
rs515135	2:21286057	*APOB*	T	C	CAD	0.031
rs312949	2:21334283	*TDRD15*	C	G	LDL;TC	0
rs6545814	2:25131316	*ADCY3*	G	A	BMI	0.0269
rs736699	2:26913930	*KCNK3*	G	A	stroke	-0.001
rs1275988	2:26914364	*KCNK3*	T	C	BP	-0.0272
rs1260326	2:27730940	*GCKR*	C	T	TC;TG	0.0037
rs780094	2:27741237	*GCKR*	C	T	T2D	-0.023
rs10203174	2:43690030	*THADA*	T	C	T2D	6.00E-04
rs35419456	2:43725965	*THADA*	A	C	T2D	0.0022
rs582384	2:45896437	*PRKCE*	A	C	CAD	0.0101
rs9309245	2:53397048	*AC010967.2*	G	C	T2D	0.0041
rs3791679	2:56096892	*EFEMP1*	A	G	WC	0.0024
rs1116357	2:57287411	*RP11-443I9.1*	G	A	T2D	0.0133
rs1861411	2:58904177	*LINC01122*	A	G	BMI	0.039
rs243019	2:60585806	*MIR4432HG*	T	C	T2D	-0.0064
rs11125936	2:62871225	*AC092155.4*	C	T	LDL	-0.037
rs2861568	2:67571721	*AC023115.4*	A	T	WHR	-0.0051
rs7568458	2:85788175	*GGCX*	A	T	CAD	0.0538
rs17678683	2:145286559	*ZEB2*	G	T	CAD	0.0666
rs7560163	2:151637936	*AC104777.4*	G	C	T2D	-0.0209
rs4377290	2:158437683	*ACVR1C*	C	T	TC	-0.0431
rs16849225	2:164906820	*AC092684.1*	T	C	BP	-0.0633
rs12999907	2:164957251	*AC092684.1*	G	A	CAD	-0.0131
rs12692735	2:165504565	*COBLL1*	T	G	T2D	-0.0288
rs840616	2:188196469	*AC007319.1*	T	C	CAD	-0.0045

© 2024 Diao T et al. *JAMA Network Open.*

rs6725887	2:203745885	*WDR12*	C	T	CAD	0.0643
rs17517928	2:216291359	*FN1*	T	C	CAD	0.0116
rs1250229	2:216304384	*FN1*	T	C	CAD	0.046
rs2571445	2:218683154	*TNS1*	A	G	CAD	0.0258
rs2972146	2:227100698	*NEU2*	G	T	CAD	-0.0073
rs2972143	2:227116365	*NEU2*	A	G	HDL	-2.00E-04
rs11677932	2:238223955	*STK25*	A	G	CAD	-0.0094
rs7616006	3:12267648	*LINC00690*	G	A	TC	-5.00E-04
rs1801282	3:12393125	*PPARG*	G	C	T2D	-0.0181
rs6804922	3:14923396	*FGD5*	G	A	CAD	-0.0539
rs748431	3:14928077	*FGD5*	T	G	CAD	-0.0027
rs1496653	3:23454790	*UBE2E2*	G	A	T2D	0.0058
rs820430	3:27548900	*FECHP1*	A	G	BP	-0.0012
rs820429	3:27549188	*FECHP1*	G	T	BP	0.0245
rs9815354	3:41912651	*ULK4*	A	G	BP	-0.0101
rs7633770	3:46688562	*SNORD77*	A	G	CAD	0.0127
rs9854454	3:48012797	*MAP4*	T	C	BP	0
rs7617773	3:48193515	*TKT*	C	T	CAD	-0.0178
rs2535633	3:52859630	*ITIH4*	G	C	BMI	0.046
rs9810888	3:53635595	*CACNA1D*	G	T	BP	0.027
rs3774472	3:53638200	*CACNA1D*	G	A	BP	-0.0013
rs9828933	3:64002897	*PSMD6*	C	T	T2D	-0.0186
rs6795735	3:64705365	*ADAMTS9-AS2*	C	T	T2D	-0.0236
rs13078807	3:85884150	*CADM2*	G	A	BMI	0.1774
rs79223353	3:123139863	*ADCY5*	A	G	T2D	-0.0056
rs17843768	3:124448385	*UMPS*	A	C	CAD	0.0169
rs17843797	3:124453022	*UMPS*	G	T	CAD	-0.0025
rs2625967	3:129267259	*H1FOO*	G	A	WHR	-1.00E-04
rs10512861	3:132257961	*NA*	T	G	CAD	-0.0287
rs667920	3:136069472	*STAG1*	G	T	CAD	-0.0307
rs6807945	3:138052754	*NME9*	C	T	CAD	0.0508
rs9818870	3:138122122	*MRAS*	T	C	CAD	0.0351
rs7610618	3:149157706	*SIAH2*	T	C	stroke	0.0063
rs1868673	3:150187314	*TSC22D2*	A	C	WC	0.0063
rs4266144	3:156852592	*SPTSSB*	C	G	CAD	-0.0398
rs12897	3:172115902	*FNDC3B*	A	G	CAD	0
rs1470579	3:185529080	*IGF2BP2*	C	A	T2D	2.00E-04
rs10513801	3:185822353	*ETV5*	G	T	BMI	-0.0691
rs17301514	3:186613409	*RPS20P14*	A	G	T2D	0.0027
rs6808574	3:187740523	*TMEM207*	T	C	T2D	-0.0129
rs73069940	4:1236502	*CTBP1*	G	C	T2D	-0.0253
rs6818397	4:3434885	*RGS12*	T	G	TG	0.0366

© 2024 Diao T et al. *JAMA Network Open.*

rs16844401	4:3449652	*HGFAC*	A	G	CAD	0.004
rs6831256	4:3473139	*DOK7*	G	A	TG	0.0035
rs4458523	4:6289986	*WFS1*	T	G	T2D	-0.0075
rs16858082	4:45175804	*NMU*	T	C	BMI	0.0414
rs871606	4:54799245	*RP11-231C18.3*	C	T	BP	-0.0166
rs17087335	4:57838583	*NOA1*	T	G	CAD	0.0507
rs12500824	4:77416627	*SHROOM3*	A	G	CAD	-1.00E-04
rs1902859	4:81157703	*RP11-576N17.4*	C	T	BP	0
rs10857147	4:81181072	*RP11-576N17.4*	T	A	CAD	0.083
rs11099493	4:82587050	*RASGEF1B*	G	A	CAD	-0.0387
rs6829822	4:86716496	*ARHGAP24*	T	G	BP	0.0323
rs1037814	4:88049850	*AFF1*	T	C	TG	0.0026
rs3775058	4:96117371	*UNC5C*	T	A	CAD	-0.0167
rs6825911	4:111381638	*ENPEP*	C	T	BP	0.0118
rs2200733	4:111710169	*RP11-119H12.3*	C	T	stroke	0.0127
rs13115759	4:113710461	*RPL7AP30*	A	T	stroke	-0.0016
rs10010670	4:113711748	*RPL7AP30*	G	A	stroke	0.0018
rs7678555	4:120909501	*RP11-170N16.1*	C	A	CAD	0.0303
rs35879803	4:146782837	*ZNF827*	A	C	CAD	0.0063
rs4593108	4:148281001	*FHDC1*	G	C	CAD	-0.0552
rs1878406	4:148393664	*EDNRA*	T	C	CAD	5.00E-04
rs17612742	4:148414651	*EDNRA*	C	T	stroke	0.0924
rs6813195	4:153520475	*FHDC1*	T	C	T2D	-0.0179
rs6825454	4:155501188	*FGA*	C	T	stroke	0.0016
rs1976041	4:156486040	*TOMM22P4*	A	G	CAD	-0.0606
rs990620	4:156507818	*TOMM22P4*	A	G	CAD	0
rs13143871	4:156619204	*GUCY1A3*	C	T	BP	-0.0067
rs72689147	4:156639888	*GUCY1A3*	T	G	CAD	-0.0626
rs7696431	4:169687725	*PALLD*	T	G	CAD	0.0227
rs10071096	5:4012694	*CTD-2287N17.1*	A	G	CAD	-0.0522
rs1508798	5:9556694	*RP11-260E18.1*	C	T	CAD	-3.00E-04
rs1173766	5:32804528	*CTD-2197M16.1*	T	C	BP	-0.0351
rs702634	5:53271420	*ARL 15.00*	G	A	T2D	-0.0075
rs459193	5:55806751	*C5orf67*	A	G	T2D	-0.0253
rs3936511	5:55860781	*C5orf67*	G	A	CAD	0.005
rs832552	5:56113850	*MAP3K1*	T	G	CAD	3.00E-04
rs6871667	5:74604742	*JMY*	G	A	TC;LDL	0.0059
rs191835914	5:74646765	*HMGCR*	C	A	LDL	-0.0996
rs3846663	5:74655726	*HMGCR*	C	T	TC;LDL	-0.0343
rs888789	5:74988369	*POC5*	A	G	BMI	0.0142
rs6878122	5:76427311	*NA*	G	A	T2D	0.0049
rs261967	5:95850250	*CTD-2337A12.1*	C	A	BMI	0.0411

© 2024 Diao T et al. *JAMA Network Open.*

rs10064156	5:102413873	*RP11-231G15.1*	C	T	T2D	0.0082
rs11957829	5:121515195	*CTC-441N14.4*	G	A	stroke	-0.0014
rs13359291	5:122476457	*PRDM6*	G	A	BP	0.023
rs10051787	5:122732236	*CEP120*	T	C	WC	0.0089
rs273909	5:131667353	*SLC22A4*	G	A	CAD	-0.0025
rs246600	5:142516897	*ARHGAP26*	T	C	CAD	-7.00E-04
rs9687065	5:148391140	*SH3TC2*	G	A	BP	-0.026
rs6882076	5:156390297	*TIMD4*	T	C	TG;LDL;TC	-0.0235
rs351855	5:176520243	*FGFR4*	A	G	WHR	-0.0042
rs634501	5:180218668	*MGAT1*	A	G	HDL	0.0055
rs78169666	6:1349830	*RP11-157J24.2*	C	A	stroke	-0.0182
rs1334576	6:7211818	*RREB1*	G	A	WHR	-0.0024
rs9505118	6:7290437	*SSR1*	G	A	T2D	0.0187
rs9349379	6:12903957	*PHACTR1*	A	G	CAD	-0.1669
rs6909574	6:22606773	*ZFP57*	G	A	CAD	0.0023
rs6909752	6:22612629	*ZFP57*	A	G	CAD	0.0678
rs1799945	6:26091179	*HFE*	G	C	BP	0.0336
rs130071	6:31116210	*CCHCR1*	A	G	TG	0.0713
rs3130501	6:31136453	*POU5F1*	A	G	T2D	0.0255
rs9357121	6:31240479	*HLA-B*	G	T	TC;LDL	-0.0847
rs9266359	6:31332739	*DHFRP2*	T	C	BP	-0.0498
rs115696548	6:31351764	*HLA-S*	C	T	CAD	0.0644
rs2021783	6:32044851	*TNXB*	T	C	BP	-0.0447
rs10947231	6:32054346	*TNXB*	A	C	BP	-3.00E-04
rs9268402	6:32341353	*C6orf10*	A	G	CAD	-0.0327
rs3129853	6:32398648	*TBC1D22B*	A	G	TG	0.0692
rs4713766	6:34244245	*NUDT3*	A	C	BMI	-0.0024
rs17609940	6:35034800	*ANKS1A*	C	G	CAD	-0.0048
rs1321309	6:36638636	*LAP3P2*	A	G	CAD	0.0416
rs9470794	6:38106844	*ZFAND3*	C	T	T2D	0.0232
rs56336142	6:39134099	*ACTG1P9*	C	T	CAD	-0.0451
rs1535500	6:39284050	*KCNK16*	T	G	T2D	0.0199
rs16896398	6:43262704	*SLC22A7*	T	A	stroke	0.0204
rs1563788	6:43308363	*ZNF318*	T	C	BP	-0.0115
rs6905288	6:43758873	*VEGFA*	G	A	HDL;CAD;TG	-0.0376
rs556621	6:44594159	*ACTG1P9*	G	T	stroke	-0.0168
rs9473924	6:50834157	*RP11-228O6.2*	T	G	BMI	0.0215
rs9367716	6:57160572	*RNU7-66P*	G	T	CAD	0.0277
rs4613862	6:82612271	*RP11-379B8.1*	C	A	CAD	-0.0272
rs9390698	6:101296389	*ASCC3*	A	G	LDL;TC	0.0239
rs884366	6:109574095	*CCDC162P*	A	G	HDL	0.0106

© 2024 Diao T et al. *JAMA Network Open.*

rs13209747	6:127115454	*LOC105377992 - LOC105377991*	T	C	BP	0.0275
rs12202017	6:134173151	*TARID*	G	A	CAD	0
rs12524865	6:134196674	*TARID*	A	C	CAD	-0.0745
rs9376090	6:135411228	*HBS1L*	C	T	TC	-0.0204
rs17080091	6:150997401	*PLEKHG1*	T	C	CAD	-0.0012
rs17080102	6:151004770	*PLEKHG1*	C	G	BP	-0.1122
rs368123	6:160719593	*NA*	G	A	WC	-0.003
rs3120140	6:160738831	*NA*	A	G	CAD	0.0269
rs10455782	6:160839350	*SLC22A3*	T	C	CAD	0.0127
rs376563	6:160851766	*SLC22A3*	T	C	CAD	2.00E-04
rs12214416	6:160910517	*LPAL2*	A	T	CAD	0.187
rs73596816	6:161017363	*LPA*	A	G	CAD	0.0015
rs7770628	6:161018174	*LPA*	C	T	TC;LDL	0.1128
rs12175867	6:161019138	*LPA*	C	T	CAD	-0.0252
rs10267593	7:1937261	*MAD1L1*	A	G	CAD	-0.0388
rs702485	7:6449272	*NA*	A	G	HDL	0.0333
rs4724806	7:6487131	*DAGLB*	G	C	CAD	-2.00E-04
rs11509880	7:12261911	*TMEM106B*	G	A	CAD	-0.0146
rs6960043	7:15052860	*GTF3AP5*	C	T	T2D	0.0186
rs4142995	7:17919258	*SNX13*	G	T	HDL	0.0012
rs2107595	7:19049388	*HDAC9*	A	G	CAD;stroke	0.06
rs12535846	7:25861072	*AC003090.1*	A	G	WHR	0
rs4719841	7:25997536	*MIR148A*	A	G	TG	0.023
rs4722766	7:28235808	*JAZF1-AS1*	G	C	T2D	3.00E-04
rs4302748	7:36191699	*EEPD1*	A	G	LDL	0.0281
rs10278336	7:44245363	*YKT6*	G	A	T2D	-0.0224
rs2107732	7:45077978	*CCM2*	A	G	CAD	-0.0012
rs10260816	7:46010100	*LOC102723446*	C	G	BP	-0.0181
rs4917014	7:50305863	*AC020743.3*	G	T	HDL	-0.0262
rs17145738	7:72982874	*TBL2*	T	C	TG;HDL	0
rs35332062	7:73012042	*MLXIPL*	A	G	TG	0.0293
rs148910227	7:80302116	*CD36*	T	C	HDL	-0.3211
rs42039	7:92244422	*CDK6*	T	C	stroke	-0.0032
rs17477177	7:106411858	*CTB-111H14.1*	C	T	BP	0.0404
rs10953541	7:107244545	*BCAP29*	T	C	CAD	-0.0239
rs975722	7:117332914	*CFTR*	G	A	CAD	0.0299
rs806215	7:127237312	*FSCN3*	T	C	T2D	-0.0334
rs4731420	7:127863295	*MIR129-1*	C	G	T2D	-0.0067
rs11556924	7:129663496	*ZC3HC1*	T	C	CAD	-0.1089
rs7810507	7:130437476	*KLF14*	A	G	WHR	0.0315

© 2024 Diao T et al. *JAMA Network Open.*

rs13233731	7:130437689	*KLF14*	A	G	T2D	-0.0051
rs10237377	7:139757136	*PARP12*	G	T	CAD	0.0287
rs17150703	8:9745798	*LINC00599*	A	G	BMI	0
rs1495741	8:18272881	*NAT2*	A	G	TC;TG	-0.003
rs6997340	8:18286997	*NAT2*	C	T	CAD	-0.0118
rs13702	8:19824492	*LPL*	C	T	HDL	-4.00E-04
rs10096633	8:19830921	*LPL*	T	C	HDL;TG	-0.0764
rs10503675	8:20072831	*ATP6V1B2*	G	A	CAD	0.0108
rs6984210	8:22033615	*BMP1*	G	C	CAD	0.0064
rs12549902	8:41509259	*NKX6-3*	A	G	T2D	0.0094
rs13277801	8:59353534	*UBXN2B*	C	T	LDL;TC	0
rs2081687	8:59388565	*CYP7A1*	T	C	TG;TC;LDL	0.0046
rs12679556	8:72514228	*RP11-1102P16.1*	T	G	WHR	-0.0023
rs4735692	8:76615663	*AC016194.1*	A	G	BMI	0.0329
rs896854	8:95960511	*NDUFAF6*	T	C	T2D	0.0453
rs10093110	8:106565414	*ZFPM2*	A	G	CAD	-0.0174
rs2245019	8:116622906	*TRPS1*	C	A	HDL	0.0031
rs13266634	8:118184783	*SLC30A8*	T	C	T2D	-0.0134
rs2954029	8:126490972	*RP11-136O12.2*	A	T	CAD	0.0224
rs11136341	8:145043543	*PLEC*	G	A	TC;LDL	0.0061
rs11787335	8:145044104	*PLEC*	T	C	TC;LDL	0
rs16927668	9:8369533	*PTPRD*	C	T	T2D	-0.0014
rs10757274	9:22096055	*CDKN2B-AS1*	G	A	CAD	0.1712
rs7859727	9:22102165	*CDKN2B-AS1*	C	T	stroke	-6.00E-04
rs2383208	9:22132076	*CDKN2B-AS1*	G	A	T2D	0.0269
rs1575972	9:22301092	*RP11-408N14.1*	A	T	T2D	-0.0171
rs10968576	9:28414339	*LINGO2*	G	A	BMI	0.0162
rs16933812	9:36969205	*PAX5*	G	T	BMI	-1.00E-04
rs11142387	9:72998332	*KLF9*	C	A	BMI	0.0168
rs17791513	9:81905590	*CHCHD2P9*	G	A	T2D	-0.0099
rs2796441	9:84308948	*TLE1*	G	A	T2D	0.0103
rs1211166	9:87285992	*NTRK2*	G	A	BMI	-0.0027
rs10820405	9:106010237	*LINC01492*	A	G	stroke	0
rs2230808	9:107562804	*ABCA1*	T	C	TC;HDL	0.0028
rs2066714	9:107586753	*ABCA1*	T	C	HDL;TC	-0.044
rs1883025	9:107664301	*ABCA1*	T	C	HDL;TC	0
rs2575876	9:107665739	*ABCA1*	A	G	HDL;TC	-0.047
rs3887137	9:107698612	*RP11-217B7.2*	T	C	WHR	0.0186
rs944172	9:110517794	*AL162389.1*	C	T	CAD	0.0334
rs4836831	9:123496570	*AHCYP2*	C	T	WC	0.0125
rs885150	9:124420173	*DAB2IP*	C	T	CAD	0.0402

© 2024 Diao T et al. *JAMA Network Open.*

rs507666	9:136149399	*ABO*	A	G	TC;LDL	0.0017
rs579459	9:136154168	*ABO*	C	T	CAD;TC;LDL	0.0038
rs635634	9:136155000	*ABO*	T	C	stroke	0.0642
rs11787792	9:139252148	*GPSM1*	G	A	T2D	-0.0602
rs1832007	10:5254847	*AKR1C4*	G	A	TG	-9.00E-04
rs7916879	10:12300790	*RN7SL232P*	G	A	CAD	-0.0223
rs11257655	10:12307894	*RN7SL232P*	C	T	T2D	-4.00E-04
rs2487928	10:30323892	*KIAA1462*	A	G	CAD	0.0021
rs1870634	10:44480811	*LINC00841*	T	G	CAD	-0.0449
rs7897379	10:65301725	*REEP3*	C	T	TG	0.0062
rs12242953	10:70865342	*SRGN*	A	G	T2D	0.0039
rs7901016	10:74637326	*MCU*	C	T	LDL	-0.0707
rs12571751	10:80942631	*ZMIZ1*	G	A	T2D	-0.0081
rs1412444	10:91002927	*LIPA*	T	C	CAD	0.0697
rs7087591	10:94473629	*Y_RNA*	G	A	T2D	0.0201
rs2068888	10:94839642	*CYP26A1*	G	A	TG;HDL	0.0272
rs9663362	10:95895177	*PLCE1*	G	C	BP	-0.009
rs7917772	10:104487443	*SFXN2*	A	G	WHR	0.0015
rs4409766	10:104616663	*BORCS7*	C	T	BP	-0.0733
rs2295786	10:105616482	*SH3PXD2A*	A	T	stroke	5.00E-04
rs4918072	10:105693644	*STN 1.00*	A	G	CAD	0.0176
rs2297991	10:113913222	*GPAM*	T	C	HDL;TC	0.0238
rs7903146	10:114758349	*TCF7L2*	T	C	T2D	0.027
rs11196288	10:115057443	*RNU7-165P*	G	A	stroke	0
rs2782980	10:115781527	*ADRB1*	T	C	BP	-0.0285
rs10886471	10:121149403	*GRK5*	T	C	T2D	-0.0017
rs4752700	10:124237612	*HTRA1*	G	A	CAD	0.0281
rs2334499	11:1696849	*FAM99B*	C	T	T2D	-0.0023
rs7107784	11:2215089	*MIR4686*	G	A	T2D	0.0133
rs117601636	11:2642037	*KCNQ1*	G	A	T2D	-0.0226
rs2237892	11:2839751	*KCNQ1*	T	C	BMI	-5.00E-04
rs2237896	11:2858440	*KCNQ1*	A	G	T2D	-0.0265
rs11601507	11:5701074	*TRIM5*	A	C	CAD	0.0663
rs10160804	11:8600240	*STK33*	A	C	BMI	0.0036
rs93138	11:9759713	*SWAP70*	G	T	CAD	0.0171
rs173396	11:9759918	*SWAP70*	A	G	CAD	0.0224
rs1351525	11:13301548	*ARNTL*	A	T	CAD	-0.0407
rs4336994	11:16302494	*SOX6*	G	A	BP	0.013
rs4757391	11:16302939	*SOX6*	C	T	BP	-0.0024
rs5215	11:17408630	*KCNJ11*	C	T	T2D	0.0259
rs11030104	11:27684517	*BDNF*	G	A	BMI	-0.0696

rs7116641	11:43696917	*RP11-472I20.4*	G	T	CAD	0.0038
rs326214	11:47298360	*MADD*	A	G	HDL	0.0048
rs11604680	11:47457539	*RAPSN*	G	A	BMI	0.0143
rs751984	11:61278246	*LRRC10B*	C	T	BP	-0.0454
rs174546	11:61569830	*FADS1*	T	C	HDL;TG;TC	0.0054
rs174547	11:61570783	*FADS1*	C	T	HDL;TC;TG	0.0086
rs12801636	11:65391317	*PCNX3*	A	G	CAD;HDL	-0.065
rs1552224	11:72433098	*ARAP1*	C	A	T2D	-0.0457
rs590121	11:75274150	*SERPINH1*	T	G	CAD	0.0446
rs499974	11:75455021	*RN7SL786P*	A	C	HDL	0.0215
rs10830963	11:92708710	*MTNR1B*	G	C	T2D	0.0017
rs633185	11:100593538	*ARHGAP42*	C	G	BP	0.0449
rs7947761	11:100624599	*ARHGAP42*	G	A	CAD	4.00E-04
rs7120712	11:100638002	*ARHGAP42*	A	G	CAD	0.0627
rs660599	11:102729757	*MMP12*	A	G	stroke	0
rs2128739	11:103673277	*RP11-563P16.1*	C	A	CAD	-0.0865
rs180327	11:116623659	*BUD13*	C	T	HDL;TG	-2.00E-04
rs964184	11:116648917	*ZPR1*	G	C	CAD	0.0079
rs2075291	11:116661392	*APOA5*	A	C	TG;HDL	0.1241
rs651821	11:116662579	*APOA5*	C	T	TC;TG;HDL	0.0622
rs12718465	11:116707736	*APOA1*	T	C	HDL	0.0568
rs17122278	11:118449370	*ARCN1*	G	A	TC	0.0069
rs17135399	11:126218541	*DCPS*	G	A	LDL	0.0355
rs4766228	12:4363420	*CCND2-AS1*	G	A	T2D	-0.0113
rs4765773	12:4366536	*CCND2-AS1*	T	C	T2D	-0.0052
rs11838267	12:7175872	*C1S*	C	T	CAD	-0.0352
rs4883263	12:7649484	*CD163*	T	C	HDL	-0.0187
rs4883201	12:9082581	*PHC1*	G	A	TC	-0.0036
rs7980458	12:20162571	*RP11-405A12.2*	G	T	BP	0.0576
rs3861086	12:20218869	*RP11-664H17.1*	T	C	CAD	0.0011
rs7304841	12:20577593	*PDE3A*	C	A	stroke	-0.0061
rs1027087	12:26470850	*RP11-283G6.4*	T	A	WHR	0.0113
rs10842992	12:27963839	*RN7SKP15*	C	T	T2D	-0.0216
rs80234489	12:31441179	*FAM60A*	C	A	T2D	-0.0193
rs897057	12:50266279	*FAIM2*	T	C	BMI	-0.0345
rs7306523	12:53393964	*EIF4B*	G	A	LDL;TC	0.0149
rs11170820	12:54513915	*FLJ12825*	G	C	CAD	0.0933
rs3809128	12:56709919	*CNPY2*	T	C	WC	0.0138
rs2261181	12:66212318	*RPSAP52*	T	C	T2D	0.0058
rs7955901	12:71433293	*CTD-2021H9.2*	T	C	T2D	-0.0109
rs17249754	12:90060586	*ATP2B1*	A	G	BP	0.0503
rs7136259	12:90081188	*ATP2B1*	T	C	CAD	-5.00E-04

© 2024 Diao T et al. *JAMA Network Open.*

rs11107829	12:95366558	*NDUFA12*	C	A	CAD	-0.0791
rs4923678	12:95368163	*NDUFA12*	G	A	CAD	-0.0031
rs34008534	12:95381088	*NDUFA12*	G	A	CAD	-4.00E-04
rs7965082	12:100800193	*SLC17A8*	T	C	LDL;TC	-0.0035
rs2075260	12:109696838	*ACACB*	G	A	TG	-0.0231
rs9593	12:109994870	*MMAB*	T	A	HDL	0.0234
rs7134594	12:110000193	*MMAB*	T	C	HDL	0.0029
rs12229654	12:111414461	*LINC01405*	G	T	BMI	-0.0068
rs671	12:112241766	*ALDH2*	A	G	HDL	0.1233
rs11066280	12:112817783	*HECTD4*	A	T	CAD	0
rs35444	12:115552437	*RP11-25E2.1*	G	A	BP;stroke	-0.0477
rs35432	12:115555530	*RP11-25E2.1*	T	C	BP	0
rs11067762	12:116198214	*RP11-110L15.1*	A	G	BP	-0.0433
rs11067763	12:116198341	*RP11-110L15.1*	G	A	BP	0.0018
rs1169288	12:121416650	*HNF1A*	C	A	LDL;TC	0.0189
rs2244608	12:121416988	*HNF1A*	G	A	CAD	0.0032
rs55783344	12:121432299	*HNF1A*	T	C	T2D	0.0074
rs2258287	12:121454313	*NA*	C	A	CAD	0.0059
rs3213545	12:121471337	*OASL*	A	G	CAD	8.00E-04
rs4275659	12:123447928	*ABCB9*	T	C	T2D	-0.0022
rs10773003	12:123775127	*SBNO1*	A	G	HDL	-0.0244
rs838880	12:125261593	*SCARB1*	T	C	HDL	0.0094
rs11057830	12:125307053	*SCARB1*	A	G	CAD	0.0227
rs10846744	12:125312425	*SCARB1*	G	C	CAD	-0.0283
rs9552911	13:23864657	*SGCG*	A	G	T2D	-0.0187
rs9512699	13:28029896	*MTIF3*	G	A	BMI	0.0043
rs9319428	13:28973621	*FLT1*	A	G	CAD	0.0568
rs9534262	13:32936646	*BRCA2*	T	C	LDL	0.0341
rs9591012	13:33058333	*N4BP2L2*	A	G	CAD	0.0014
rs1467605	13:47211861	*LRCH1*	A	C	stroke	-2.00E-04
rs9568867	13:54107352	*RP11-384G23.1*	A	G	BMI	0.0561
rs1359790	13:80717156	*RP11-470M1.2*	A	G	T2D	-0.0103
rs7989336	13:97017548	*HS6ST3*	A	G	BMI	0.0104
rs11838776	13:111040681	*COL4A2*	A	G	CAD	0.0831
rs1317507	13:113631780	*NA*	A	C	CAD	0.0296
rs2415317	14:36609678	*LINC00609*	A	G	stroke	0
rs1982963	14:52509101	*NID2*	G	A	WHR	0.0124
rs2145598	14:58794001	*ARID4A*	A	G	CAD	-0.0196
rs2268617	14:75608728	*TMED10*	G	A	CAD	-0.044
rs11624704	14:78786077	*NRXN3*	C	A	BMI	0.0029
rs10139550	14:100145710	*HHIPL1*	G	C	CAD	0.0368
rs7403531	15:38822905	*NA*	T	C	T2D	0.0079

© 2024 Diao T et al. *JAMA Network Open.*

rs67839313	15:40619724	*C15orf52*	C	T	T2D	0.0097
rs4471613	15:58551694	*ALDH1A2, AQP9, LIPC*	A	G	stroke	-4.00E-04
rs1532085	15:58683366	*NA*	A	G	TC;TG;HDL	0.0046
rs1077834	15:58723479	*LIPC*	C	T	HDL;TC	0
rs1800588	15:58723675	*LIPC*	T	C	TG;TC;HDL	0.0256
rs1436953	15:62414014	*NPM1P47*	T	C	T2D	-0.0094
rs6494488	15:65024204	*RBPMS2*	G	A	CAD	-0.067
rs4777561	15:65064585	*RBPMS2*	T	C	CAD	-3.00E-04
rs56062135	15:67455630	*SMAD3*	T	C	CAD	-0.1237
rs4776970	15:68080886	*MAP2K5*	A	T	BMI	0.032
rs1378942	15:75077367	*CSK*	A	C	BP	-0.0431
rs7178572	15:77747190	*HMG20A*	G	A	T2D	0.0345
rs4468572	15:79124475	*MORF4L1*	T	C	CAD	-0.0807
rs11634397	15:80432222	*ZFAND6*	G	A	T2D	2.00E-04
rs8030379	15:84590910	*ADAMTSL3*	G	A	WC	-0.0183
rs8042271	15:89574218	*RP11-326A19.2*	A	G	CAD	-0.0722
rs2028299	15:90374257	*AP3S2*	C	A	T2D	0.0074
rs4932370	15:91404705	*RN7SL363P*	A	G	stroke	0
rs17514846	15:91416550	*FURIN*	A	C	CAD	0.0942
rs1029420	15:91441086	*MAN2A2*	C	T	BP	-0.0012
rs79548680	15:91505779	*RCCD1*	C	G	T2D	0.0069
rs2531995	16:4013467	*ADCY9*	T	C	BMI	0.0286
rs12597579	16:20257867	*GP2*	T	C	BMI	-0.0772
rs4788102	16:28873398	*SH2B1*	A	G	BMI	0.0422
rs1421085	16:53800954	*FTO*	C	T	T2D	0.0333
rs1558902	16:53803574	*FTO*	A	T	BMI	0.0727
rs247616	16:56989590	*AC012181.1*	T	C	HDL;TC	0.0077
rs7499892	16:57006590	*CETP*	T	C	TG;HDL;LDL	0.0186
rs2303790	16:57017292	*CETP*	G	A	HDL	-0.0485
rs2292318	16:67985706	*SLC12A4*	T	C	HDL	0
rs3785100	16:67997920	*SLC12A4*	C	T	HDL	0.0391
rs17358402	16:71967927	*PKD1L3*	T	C	TC;LDL	0.0642
rs7185272	16:72013797	*PKD1L3*	G	C	LDL;TC	-0.0408
rs12927205	16:72025077	*PKD1L3*	G	A	LDL;TC	-0.0098
rs2000999	16:72108093	*HPR*	A	G	TC;LDL	0.0572
rs1050362	16:72130815	*DHX38*	A	C	CAD	2.00E-04
rs7193343	16:73029160	*ZFHX3*	C	T	stroke	-4.00E-04
rs12932445	16:73069888	*ZFHX3*	C	T	stroke	-0.0288
rs7202877	16:75247245	*CTRB1*	G	T	T2D	-0.0267
rs7206541	16:75436713	*CFDP1*	A	T	CAD	-0.0594

© 2024 Diao T et al. *JAMA Network Open.*

rs1424233	16:79682751	*AC009159.1*	C	T	BMI	1.00E-04
rs2925979	16:81534790	*CMIP*	T	C	HDL	0.0238
rs7199941	16:81906423	*PLCG2*	A	G	CAD	0.0351
rs7500448	16:83045790	*CDH13*	G	A	CAD	-0.036
rs12445022	16:87575332	*RP11-482M8.1*	A	G	stroke	-0.0199
rs216172	17:2126504	*SMG6*	C	G	CAD	0.0361
rs391300	17:2216258	*SRR*	T	C	T2D	0.0078
rs13342232	17:6945940	*SLC16A11*	G	A	T2D	0.0169
rs12936587	17:17543722	*RP11-524F11.3*	A	G	CAD	-0.0155
rs13723	17:27941886	*CORO6*	G	A	CAD	0.0302
rs76954792	17:30033514	*RP11-805L22.1*	T	C	CAD	0.0504
rs7208487	17:37543449	*FBXL20*	G	T	HDL	0.0251
rs2074158	17:40257163	*DHX58*	C	T	CAD	0.0044
rs12603327	17:40290552	*CTD-2132N18.3*	C	T	CAD	0.0518
rs12946454	17:43208121	*PLCD3*	T	A	BP	0.0037
rs17608766	17:45013271	*GOSR2*	C	T	CAD	8.00E-04
rs9299	17:46669430	*HOXB5*	C	T	BMI	-0.0285
rs7405452	17:46674670	*HOXB6*	T	C	BP	-0.0171
rs46522	17:46988597	*UBE2Z*	C	T	CAD	-0.0327
rs7213603	17:59003189	*BCAS3*	C	T	CAD	0.0122
rs7225581	17:59017813	*BCAS3*	A	T	CAD	0.054
rs2240736	17:59485393	*TBX2*	T	C	BP	0.0205
rs1867624	17:62387091	*RPL31P57*	C	T	CAD	0
rs9892152	17:62401965	*RPL31P57*	T	C	CAD	-0.0515
rs4148008	17:66875294	*ABCA8*	G	C	HDL	0.0205
rs9916693	17:67138563	*ABCA6*	A	T	LDL;TC	0
rs12453914	17:67138878	*ABCA6*	A	C	LDL;TC	0.0021
rs11077501	17:68526061	*RP11-1058G23.1*	C	T	WHR	0.0163
rs4129767	17:76403984	*PGS1*	A	G	HDL	0.0175
rs7503807	17:78591111	*RPTOR*	C	A	BMI	-0.0245
rs8090011	18:7068462	*NA*	C	G	T2D	0.0461
rs7228667	18:20015273	*RP11-863N1.4*	C	T	CAD	0
rs12607689	18:20016299	*RP11-863N1.4*	T	G	CAD	-2.00E-04
rs4410190	18:20020200	*RP11-863N1.4*	C	T	CAD	-0.0531
rs2000813	18:47093864	*LIPG*	T	C	HDL	0.0044
rs12970066	18:47107152	*LIPG*	G	C	HDL	-0.0312
rs4939883	18:47167214	*SMUG1P1*	T	C	HDL;TC	3.00E-04
rs2156552	18:47181668	*SMUG1P1*	A	T	HDL	0.0179
rs11660468	18:47209143	*SMUG1P1*	T	C	CAD	0.0039
rs35337492	18:47210824	*SMUG1P1*	A	G	CAD	0.0157
rs663129	18:57838401	*RNU4-17P*	A	G	CAD	0.088

© 2024 Diao T et al. *JAMA Network Open.*

rs740406	19:2232221	*NA*	G	A	BP	-0.0086
rs2229383	19:10794630	*ILF3-SLC44A2*	G	T	stroke	-0.0352
rs7258189	19:11169947	*SMARCA4*	C	T	stroke	0.0018
rs56289821	19:11188247	*LDLR*	A	G	CAD	0.2471
rs7258950	19:11250139	*SPC24*	A	G	LDL;TC	-0.008
rs11557092	19:11257018	*SPC24*	T	C	TC;LDL	-0.0733
rs737337	19:11347493	*DOCK6*	C	T	LDL;HDL;TC	-0.0078
rs73015714	19:17855763	*FCHO1*	G	C	CAD	0.0755
rs58542926	19:19379549	*TM6SF2*	T	C	TC;TG;LDL	0.0066
rs10401969	19:19407718	*SUGP1*	C	T	T2D	-0.0085
rs16967013	19:32859514	*ZNF507*	G	C	CAD	0.002
rs29941	19:34309532	*KCTD15*	G	A	BMI	0.022
rs7258445	19:41855515	*TGFB1*	A	G	CAD	-0.0501
rs2075650	19:45395619	*TOMM40*	G	A	CAD;BMI	-3.00E-04
rs157582	19:45396219	*TOMM40*	T	C	TG	0
rs769449	19:45410002	*APOE*	A	G	TG;HDL;LDL;TC	-0.0047
rs72654473	19:45414399	*APOC1*	A	C	TC;TG	-0.2052
rs439401	19:45414451	*APOC1*	C	T	HDL;TC;TG	0
rs4420638	19:45422946	*APOC1*	G	A	CAD	0.0853
rs8108269	19:46158513	*RN7SL836P*	G	T	T2D	0.0028
rs2302593	19:46196634	*GIPR*	G	C	CAD	-0.0019
rs3810291	19:47569003	*ZC3H4*	A	G	BMI	0.0031
rs17695224	19:52324216	*FPR3*	A	G	HDL	0.0116
rs6038557	20:6594349	*RP5-971N18.3*	G	A	WC	-0.0288
rs1887320	20:10965998	*RP11-103J8.1*	G	A	BP	-0.0381
rs2328223	20:17845921	*RIN2*	C	A	LDL	0.0108
rs867186	20:33764554	*PROCR*	G	A	CAD	-0.0554
rs4911495	20:33971978	*UQCC1*	C	A	WHR	-0.0134
rs6065311	20:39724338	*TOP 1.00*	T	C	LDL	-0.0518
rs6093446	20:39780932	*PLCG1*	A	G	CAD	0.0037
rs4812829	20:42989267	*HNF4A*	A	G	T2D	0.023
rs3827066	20:44586023	*ZNF335*	T	C	CAD	0.0664
rs16990971	20:44601293	*ZNF335*	G	A	TG	-0.0433
rs13041126	20:51092996	*LINC01524*	C	T	BMI	-0.0097
rs2057291	20:57472043	*GNAS*	A	G	CAD;WC	0.0496
rs11911017	21:30567941	*BACH1*	T	G	CAD	0.0104
rs181359	22:21928641	*UBE2L3*	A	G	HDL	-0.0021
rs181360	22:21928916	*UBE2L3*	G	T	HDL	-0.0287
rs16999793	22:24656875	*POM121L9P*	C	G	CAD	-0.0687
rs4821382	22:35638115	*SCUBE1*	G	C	WHR	-0.002

rs5996074	22:42236337	*SREBF2*	G	A	BMI	0.0023
rs738409	22:44324727	*PNPLA3*	G	C	TG	-0.027
rs1800234	22:46615880	*PPARA*	C	T	TG	0

Abbreviations: CHD, coronary heart disease; PRS, polygenic risk score; CAD, coronary artery disease; HDL-C, high density lipoprotein cholesterol; LDL-C, low density lipoprotein cholesterol; TC, total cholesterol; TG, triglycerides; BMI, body mass index; WHR, waist to hip ratio; WC, waist circumference; BP, blood pressure; T2D, type 2 diabetes.

© 2024 Diao T et al. *JAMA Network Open.*

eTable 2. Genetic Variants Included in the Stroke PRS

Genetic variant	Chr:Pos hg19	Gene	Risk allele	Other allele	Trait	Weight
rs36096196	1:2252205	*MORN1*	T	C	CAD	0.019219
rs880315	1:10796866	*CASZ1*	C	T	BP;stroke	0.052414
rs4846049	1:11850365	*MTHFR*	G	T	BP	0.016598
rs12027135	1:25775733	*TMEM57*	T	A	TC;LDL-C	0.006393
rs61776719	1:38461319	*SF3A3*	A	C	CAD	0.011104
rs2296172	1:39835817	*MACF1*	G	A	HDL-C	0.005624
rs11205760	1:51174330	*FAF1*	T	C	T2D	0.010276
rs11206510	1:55496039	*PCSK9*	T	C	CAD	0.025281
rs7525649	1:55499156	*PCSK9*	T	C	LDL-C;TC	0.006168
rs151193009	1:55509585	*PCSK9*	C	T	LDL-C;TC	0.139165
rs9970807	1:56965664	*PLPP3*	T	C	CAD	0.00491
rs12042319	1:63049819	*DOCK7*	A	G	TC;TG	0.000925
rs995000	1:63107526	*DOCK7*	C	T	TG	0.003734
rs10889353	1:63118196	*DOCK7*	A	C	TC;TG	0.010123
rs2815752	1:72812440	*RPL31P12*	G	A	BMI	0.013035
rs1514175	1:74991644	*FPGT-TNNI3K*	A	G	BMI	0.004949
rs17381664	1:78048331	*ZZZ3*	T	C	BMI	0.021861
rs117711462	1:93159927	*EVI5*	A	G	LDL-C;TC	0.098448
rs1555543	1:96944797	*EEF1A1P11*	A	C	BMI	0.004137
rs7528419	1:109817192	*CELSR2*	G	A	CAD	7.50E-05
rs12740374	1:109817590	*CELSR2*	G	T	LDL-C;TC	0.03527
rs12037987	1:113042822	*WNT2B*	C	T	stroke	0.05473
rs10745332	1:113189053	*CAPZA1*	A	G	BP	0.015384
rs17030613	1:113190807	*CAPZA1*	C	A	BP	0.016001
rs984222	1:119503843	*TBX15*	G	C	WHR	0.020995
rs10923931	1:120517959	*NOTCH2*	T	G	T2D	0.001803
rs11810571	1:151762308	*TDRKH*	G	C	CAD	0.017231
rs67156297	1:154336716	*RP11-350G8.4*	A	G	T2D	0.017919
rs4845625	1:154422067	*IL6R*	T	C	CAD	0.002666
rs6666258	1:154814268	*KCNN3*	C	G	AF	0.00706
rs1052053	1:156202173	*PMF1-SEMA4A*	A	G	stroke	0.005164
rs2758607	1:156202759	*PMF1-BGLAP*	G	A	stroke	0.023813
rs1892094	1:169094459	*ATP1B1*	C	T	CAD	0.01956
rs1200159	1:169100241	*ATP1B1*	T	C	CAD	0.000738
rs3903239	1:170569317	*RP1-79C4.1*	G	A	AF	0.013596
rs2213732	1:172333595	*DNM3*	G	A	WHR	0.010511
rs574367	1:177873210	*SEC16B*	T	G	BMI	0.044171
rs1689800	1:182168885	*LINC01344*	G	A	HDL-C	0.005133
rs6700559	1:200646073	*RP11-92G12.3*	C	T	CAD	0.013816

© 2024 Diao T et al. *JAMA Network Open.*

rs2820315	1:201872264	*LMOD1*	C	T	CAD	0.01037
rs2819348	1:201884952	*LMOD1*	C	T	CAD	0.010745
rs60154123	1:210468999	*RP4-667H12.4*	C	T	CAD	0.000112
rs2075423	1:214154719	*PROX1*	G	T	T2D	0.010935
rs340874	1:214159256	*PROX1*	C	T	T2D	0.011132
rs2820443	1:219753509	*RP11-95P13.2*	C	T	WHR	0.006163
rs2642442	1:220973563	*NA*	T	C	TC;LDL-C	0.004286
rs17465637	1:222823529	*MIA3*	C	A	CAD	0.011236
rs67180937	1:222823743	*MIA3*	G	T	CAD	0.004491
rs2144300	1:230294916	*GALNT2*	C	T	TG	0.005569
rs699	1:230845794	*AGT*	G	A	CAD	0.011681
rs2783963	1:243501583	*SDCCAG8*	G	A	BMI	0.005338
rs12463617	2:629244	*TMEM18*	C	A	BMI	0.024811
rs1344653	2:19730845	*LINC01808*	G	A	BP	0.009267
rs16986953	2:19942473	*AC019055.1*	A	G	CAD	0.00986
rs2123536	2:19945577	*AC019055.1*	T	C	CAD	0.001809
rs13306194	2:21252534	*APOB*	G	A	LDL-C;TC;TG	0.022883
rs1367117	2:21263900	*APOB*	A	G	TC;LDL-C	0.003707
rs515135	2:21286057	*APOB*	T	C	CAD	0.003012
rs312949	2:21334283	*TDRD15*	C	G	LDL-C;TC	0.000872
rs6545814	2:25131316	*ADCY3*	A	G	BMI	0.002952
rs736699	2:26913930	*KCNK3*	A	G	stroke	0.037618
rs1275988	2:26914364	*KCNK3*	C	T	BP	0.036253
rs1260326	2:27730940	*GCKR*	T	C	TC;TG	0.005624
rs780094	2:27741237	*GCKR*	T	C	T2D	0.010075
rs10203174	2:43690030	*THADA*	C	T	T2D	0.029406
rs35419456	2:43725965	*THADA*	A	C	T2D	0.004535
rs582384	2:45896437	*PRKCE*	A	C	CAD	0.008268
rs9309245	2:53397048	*AC010967.2*	G	C	T2D	0.000136
rs3791679	2:56096892	*EFEMP1*	A	G	WC	0.044065
rs1116357	2:57287411	*RP11-443I9.1*	G	A	T2D	0.01577
rs1861411	2:58904177	*LINC01122*	A	G	BMI	0.019236
rs243019	2:60585806	*MIR4432HG*	T	C	T2D	0.001389
rs11125936	2:62871225	*AC092155.4*	T	C	LDL-C	0.008668
rs2861568	2:67571721	*AC023115.4*	T	A	WHR	0.012588
rs7568458	2:85788175	*GGCX*	T	A	CAD	0.00693
rs17678683	2:145286559	*ZEB2*	G	T	CAD	0.002869
rs7560163	2:151637936	*AC104777.4*	G	C	T2D	0.002374
rs4377290	2:158437683	*ACVR1C*	T	C	TC	0.013595
rs16849225	2:164906820	*AC092684.1*	C	T	BP	0.000664
rs12999907	2:164957251	*AC092684.1*	A	G	CAD	0.022381

© 2024 Diao T et al. *JAMA Network Open.*

rs12692735	2:165504565	*COBLL1*	G	T	T2D	0.016576
rs840616	2:188196469	*AC007319.1*	C	T	CAD	0.017683
rs6725887	2:203745885	*WDR12*	T	C	CAD	0.008833
rs17517928	2:216291359	*FN1*	C	T	CAD	0.034681
rs1250229	2:216304384	*FN1*	T	C	CAD	0.021837
rs2571445	2:218683154	*TNS1*	A	G	CAD	0.004345
rs2972146	2:227100698	*NEU2*	T	G	CAD	0.005336
rs2972143	2:227116365	*NEU2*	G	A	HDL-C	0.001733
rs11677932	2:238223955	*STK25*	G	A	CAD	0.004911
rs7616006	3:12267648	*LINC00690*	A	G	TC	0.003312
rs1801282	3:12393125	*PPARG*	C	G	T2D	0.008173
rs748431	3:14928077	*FGD5*	G	T	CAD	0.005765
rs1496653	3:23454790	*UBE2E2*	A	G	T2D	0.009312
rs820430	3:27548900	*FECHP1*	A	G	BP	0.02506
rs9815354	3:41912651	*ULK4*	G	A	BP	0.019701
rs7633770	3:46688562	*SNORD77*	G	A	CAD	0.001639
rs7617773	3:48193515	*TKT*	T	C	CAD	0.005907
rs2535633	3:52859630	*ITIH4*	G	C	BMI	0.018491
rs9810888	3:53635595	*CACNA1D*	G	T	BP	0.031267
rs9828933	3:64002897	*PSMD6*	T	C	T2D	0.00264
rs6795735	3:64705365	*ADAMTS9-AS2*	T	C	T2D	0.002674
rs13078807	3:85884150	*CADM2*	G	A	BMI	0.024834
rs79223353	3:123139863	*ADCY5*	G	A	T2D	0.014227
rs17843768	3:124448385	*UMPS*	A	C	CAD	0.014655
rs17843797	3:124453022	*UMPS*	T	G	CAD	0.001964
rs2625967	3:129267259	*H1FOO*	A	G	WHR	0.002395
rs10512861	3:132257961	*NA*	G	T	CAD	0.027265
rs667920	3:136069472	*STAG1*	G	T	CAD	0.001829
rs6807945	3:138052754	*NME9*	C	T	CAD	0.012289
rs9818870	3:138122122	*MRAS*	T	C	CAD	0.000305
rs7610618	3:149157706	*SIAH2*	C	T	stroke	0.024026
rs1868673	3:150187314	*TSC22D2*	C	A	WC	0.010409
rs4266144	3:156852592	*SPTSSB*	G	C	CAD	0.006155
rs12897	3:172115902	*FNDC3B*	G	A	CAD	0.017004
rs1470579	3:185529080	*IGF2BP2*	C	A	T2D	0.026911
rs10513801	3:185822353	*ETV5*	T	G	BMI	0.028359
rs17301514	3:186613409	*RPS20P14*	A	G	T2D	0.008706
rs6808574	3:187740523	*TMEM207*	T	C	T2D	0.025214
rs73069940	4:1236502	*CTBP1*	C	G	T2D	0.004464
rs6818397	4:3434885	*RGS12*	T	G	TG	0.009902
rs16844401	4:3449652	*HGFAC*	A	G	CAD	0.022233
rs6831256	4:3473139	*DOK7*	G	A	TG	0.014548

rs4458523	4:6289986	*WFS1*	G	T	T2D	0.031607
rs16858082	4:45175804	*NMU*	T	C	BMI	0.01899
rs871606	4:54799245	*RP11-231C18.3*	T	C	BP	0.032441
rs17087335	4:57838583	*NOA1*	T	G	CAD	0.019932
rs12500824	4:77416627	*SHROOM3*	A	G	CAD	0.011593
rs1902859	4:81157703	*RP11-576N17.4*	C	T	BP	0.025913
rs10857147	4:81181072	*RP11-576N17.4*	T	A	CAD	0.068632
rs11099493	4:82587050	*RASGEF1B*	A	G	CAD	0.011677
rs6829822	4:86716496	*ARHGAP24*	T	G	BP	0.026387
rs1037814	4:88049850	*AFF1*	T	C	TG	0.006616
rs3775058	4:96117371	*UNC5C*	A	T	CAD	0.016346
rs6825911	4:111381638	*ENPEP*	C	T	BP	0.026501
rs1448818	4:111570223	*RP11-119H12.3*	C	A	AF	0.000592
rs6817105	4:111705768	*RP11-119H12.3*	C	T	AF	0.012825
rs2200733	4:111710169	*RP11-119H12.3*	T	C	stroke	0.005903
rs13143308	4:111714419	*LINC01438*	T	G	stroke	0.009406
rs4400058	4:111716673	*LINC01438*	G	A	AF	0.006059
rs6838973	4:111765495	*RP11-119H12.3*	C	T	AF	0.023223
rs13115759	4:113710461	*RPL7AP30*	T	A	stroke	0.001226
rs10010670	4:113711748	*RPL7AP30*	G	A	stroke	0.001401
rs7678555	4:120909501	*RP11-170N16.1*	C	A	CAD	0.002327
rs4593108	4:148281001	*FHDC1*	C	G	CAD	0.015338
rs17612742	4:148414651	*EDNRA*	C	T	stroke	0.028273
rs6813195	4:153520475	*FHDC1*	C	T	T2D	0.015191
rs6825454	4:155501188	*FGA*	C	T	stroke	0.022614
rs1976041	4:156486040	*TOMM22P4*	G	A	CAD	0.017425
rs13143871	4:156619204	*GUCY1A3*	C	T	BP	0.000506
rs72689147	4:156639888	*GUCY1A3*	G	T	CAD	0.014592
rs7696431	4:169687725	*PALLD*	G	T	CAD	0.00203
rs1508798	5:9556694	*RP11-260E18.1*	T	C	CAD	0.009816
rs1173766	5:32804528	*CTD-2197M16.1*	C	T	BP	0.033919
rs702634	5:53271420	*ARL 15.00*	A	G	T2D	0.014191
rs459193	5:55806751	*C5orf67*	A	G	T2D	0.006189
rs3936511	5:55860781	*C5orf67*	G	A	CAD	0.009895
rs6871667	5:74604742	*JMY*	G	A	TC;LDL-C	0.004722
rs191835914	5:74646765	*HMGCR*	A	C	LDL-C	0.065337
rs3846663	5:74655726	*HMGCR*	T	C	TC;LDL-C	0.009906
rs888789	5:74988369	*POC5*	G	A	BMI	0.00193
rs6878122	5:76427311	*NA*	A	G	T2D	0.055228
rs261967	5:95850250	*CTD-2337A12.1*	C	A	BMI	0.013098
rs10064156	5:102413873	*RP11-231G15.1*	T	C	T2D	0.003601
rs11957829	5:121515195	*CTC-441N14.4*	G	A	stroke	0.00125

© 2024 Diao T et al. *JAMA Network Open.*

rs13359291	5:122476457	*PRDM6*	A	G	BP	0.007962
rs10051787	5:122732236	*CEP120*	T	C	WC	0.018973
rs7701094	5:124317011	*LMNB1*	C	G	BMI	0.018406
rs273909	5:131667353	*SLC22A4*	G	A	CAD	0.025162
rs246600	5:142516897	*ARHGAP26*	C	T	CAD	0.019302
rs9687065	5:148391140	*SH3TC2*	A	G	BP	0.014944
rs6882076	5:156390297	*TIMD4*	C	T	TG;LDL-C;TC	0.004726
rs351855	5:176520243	*FGFR4*	G	A	WHR	0.023237
rs634501	5:180218668	*MGAT1*	A	G	HDL-C	0.004097
rs12204590	6:1337393	*RP11-157J24.2*	A	T	stroke	0.363345
rs78169666	6:1349830	*RP11-157J24.2*	C	A	stroke	0.170064
rs9501744	6:1617143	*FOXC1*	C	T	CAD	0.011128
rs1334576	6:7211818	*RREB1*	A	G	WHR	0.006976
rs9505118	6:7290437	*SSR1*	G	A	T2D	0.007042
rs9349379	6:12903957	*PHACTR1*	G	A	CAD	0.016793
rs6909752	6:22612629	*ZFP57*	A	G	CAD	0.022304
rs1799945	6:26091179	*HFE*	G	C	BP	0.011981
rs130071	6:31116210	*CCHCR1*	A	G	TG	0.029474
rs3130501	6:31136453	*POU5F1*	G	A	T2D	0.002329
rs9357121	6:31240479	*HLA-B*	T	G	TC;LDL-C	0.026952
rs9266359	6:31332739	*DHFRP2*	C	T	BP	0.028754
rs2021783	6:32044851	*TNXB*	C	T	BP	0.01146
rs9268402	6:32341353	*C6orf10*	G	A	CAD	0.006782
rs3129853	6:32398648	*TBC1D22B*	A	G	TG	0.013553
rs4713766	6:34244245	*NUDT3*	C	A	BMI	0.007025
rs17609940	6:35034800	*ANKS1A*	C	G	CAD	0.009008
rs1321309	6:36638636	*LAP3P2*	A	G	CAD	0.013167
rs9470794	6:38106844	*ZFAND3*	C	T	T2D	0.009198
rs56336142	6:39134099	*ACTG1P9*	T	C	CAD	0.006196
rs1535500	6:39284050	*KCNK16*	T	G	T2D	0.009901
rs16896398	6:43262704	*SLC22A7*	T	A	stroke	0.041632
rs6905288	6:43758873	*VEGFA*	A	G	HDL-C;CAD;TG	0.017852
rs556621	6:44594159	*ACTG1P9*	T	G	stroke	0.013469
rs9473924	6:50834157	*RP11-228O6.2*	T	G	BMI	0.013494
rs9367716	6:57160572	*RNU7-66P*	G	T	CAD	0.005278
rs4613862	6:82612271	*RP11-379B8.1*	A	C	CAD	0.001835
rs9390698	6:101296389	*ASCC3*	A	G	LDL-C;TC	0.002508
rs884366	6:109574095	*CCDC162P*	A	G	HDL-C	0.008103
rs13216675	6:122452329	*TRMT11*	T	C	AF	0.007378

© 2024 Diao T et al. *JAMA Network Open.*

rs13209747	6:127115454	*LOC105377992 – LOC105377991*	T	C	BP	0.019474
rs12202017	6:134173151	*TARID*	G	A	CAD	0.007572
rs12524865	6:134196674	*TARID*	C	A	CAD	0.017232
rs9376090	6:135411228	*HBS1L*	C	T	TC	0.005716
rs17080091	6:150997401	*PLEKHG1*	C	T	CAD	0.023063
rs17080102	6:151004770	*PLEKHG1*	G	C	BP	0.020059
rs368123	6:160719593	*NA*	G	A	WC	0.009484
rs3120140	6:160738831	*NA*	A	G	CAD	0.004358
rs10455782	6:160839350	*SLC22A3*	T	C	CAD	0.00651
rs376563	6:160851766	*SLC22A3*	T	C	CAD	0.00762
rs12214416	6:160910517	*LPAL2*	A	T	CAD	0.029653
rs7770628	6:161018174	*LPA*	C	T	TC;LDL-C	0.029883
rs10267593	7:1937261	*MAD1L1*	G	A	CAD	0.014634
rs702485	7:6449272	*NA*	A	G	HDL-C	0.03332
rs4724806	7:6487131	*DAGLB*	C	G	CAD	0.015422
rs11509880	7:12261911	*TMEM106B*	A	G	CAD	0.008943
rs6960043	7:15052860	*GTF3AP5*	C	T	T2D	0.020004
rs4142995	7:17919258	*SNX13*	G	T	HDL-C	0.002416
rs2107595	7:19049388	*HDAC9*	A	G	CAD;stroke	0.03644
rs12535846	7:25861072	*AC003090.1*	G	A	WHR	0.001852
rs4719841	7:25997536	*MIR148A*	G	A	TG	0.00167
rs4722766	7:28235808	*JAZF1-AS1*	G	C	T2D	0.000162
rs4302748	7:36191699	*EEPD1*	A	G	LDL-C	0.004334
rs10278336	7:44245363	*YKT6*	A	G	T2D	0.012139
rs10260816	7:46010100	*LOC102723446*	C	G	BP	0.01244
rs4917014	7:50305863	*AC020743.3*	G	T	HDL-C	0.001912
rs35332062	7:73012042	*MLXIPL*	A	G	TG	0.002028
rs42039	7:92244422	*CDK6*	C	T	stroke	0.005031
rs17477177	7:106411858	*CTB-111H14.1*	C	T	BP	0.030189
rs10953541	7:107244545	*BCAP29*	C	T	CAD	0.007904
rs3807989	7:116186241	*CAV1*	G	A	AF	0.001154
rs975722	7:117332914	*CFTR*	G	A	CAD	0.017304
rs806215	7:127237312	*FSCN3*	C	T	T2D	0.01009
rs4731420	7:127863295	*MIR129-1*	C	G	T2D	0.016028
rs11556924	7:129663496	*ZC3HC1*	C	T	CAD	0.01246
rs7810507	7:130437476	*KLF14*	A	G	WHR	0.01256
rs13233731	7:130437689	*KLF14*	G	A	T2D	0.014749
rs10237377	7:139757136	*PARP12*	G	T	CAD	0.012351
rs17150703	8:9745798	*LINC00599*	G	A	BMI	0.001752
rs1495741	8:18272881	*NAT2*	G	A	TC;TG	0.014478

© 2024 Diao T et al. *JAMA Network Open.*

rs6997340	8:18286997	*NAT2*	T	C	CAD	0.006814
rs10096633	8:19830921	*LPL*	C	T	HDL-C; TG	0.021775
rs6984210	8:22033615	*BMP1*	G	C	CAD	0.005012
rs12549902	8:41509259	*NKX6-3*	A	G	T2D	0.003175
rs13277801	8:59353534	*UBXN2B*	T	C	LDL-C;TC	0.005375
rs2081687	8:59388565	*CYP7A1*	T	C	TG;TC;LDL-C	0.005005
rs12679556	8:72514228	*RP11-1102P16.1*	G	T	WHR	0.004965
rs4735692	8:76615663	*AC016194.1*	A	G	BMI	0.01738
rs896854	8:95960511	*NDUFAF6*	T	C	T2D	0.010004
rs10093110	8:106565414	*ZFPM2*	G	A	CAD	0.019755
rs2245019	8:116622906	*TRPS1*	A	C	HDL-C	0.008681
rs13266634	8:118184783	*SLC30A8*	C	T	T2D	0.015439
rs2954029	8:126490972	*RP11-136O12.2*	A	T	CAD	0.009622
rs11136341	8:145043543	*PLEC*	A	G	TC;LDL-C	0.005552
rs16927668	9:8369533	*PTPRD*	T	C	T2D	0.002096
rs10757274	9:22096055	*CDKN2B-AS1*	G	A	CAD	0.033065
rs7859727	9:22102165	*CDKN2B-AS1*	T	C	stroke	0.011696
rs1333042	9:22103813	*CDKN2B-AS1*	A	G	CAD	0.000564
rs2383208	9:22132076	*CDKN2B-AS1*	A	G	T2D	0.017616
rs1575972	9:22301092	*RP11-408N14.1*	T	A	T2D	0.023269
rs10968576	9:28414339	*LINGO2*	G	A	BMI	0.011499
rs16933812	9:36969205	*PAX5*	G	T	BMI	0.001819
rs11142387	9:72998332	*KLF9*	A	C	BMI	0.002104
rs17791513	9:81905590	*CHCHD2P9*	A	G	T2D	0.000414
rs2796441	9:84308948	*TLE1*	G	A	T2D	0.003599
rs1211166	9:87285992	*NTRK2*	G	A	BMI	0.000426
rs10821415	9:97713459	*C9orf3*	C	A	AF	0.000268
rs10820405	9:106010237	*LINC01492*	A	G	stroke	0.004425
rs2230808	9:107562804	*ABCA1*	C	T	TC;HDL-C	0.005379
rs2066714	9:107586753	*ABCA1*	C	T	HDL-C;TC	0.005754
rs1883025	9:107664301	*ABCA1*	C	T	HDL-C;TC	0.009567
rs2575876	9:107665739	*ABCA1*	G	A	HDL-C;TC	0.012713
rs3887137	9:107698612	*RP11-217B7.2*	T	C	WHR	0.001836
rs944172	9:110517794	*AL162389.1*	C	T	CAD	0.005883
rs4836831	9:123496570	*AHCYP2*	C	T	WC	0.023306
rs885150	9:124420173	*DAB2IP*	C	T	CAD	0.012126
rs2519093	9:136141870	*ABO*	T	C	CAD	3.80E-05
rs507666	9:136149399	*ABO*	A	G	TC;LDL-C	0.007558
rs579459	9:136154168	*ABO*	C	T	CAD;TC;LDL-C	0.002391

© 2024 Diao T et al. *JAMA Network Open.*

rs11787792	9:139252148	*GPSM1*	A	G	T2D	0.029986
rs1832007	10:5254847	*AKR1C4*	A	G	TG	0.002968
rs7916879	10:12300790	*RN7SL232P*	G	A	CAD	0.000586
rs11257655	10:12307894	*RN7SL232P*	T	C	T2D	0.011323
rs2487928	10:30323892	*KIAA1462*	A	G	CAD	0.008167
rs1870634	10:44480811	*LINC00841*	T	G	CAD	0.00423
rs7897379	10:65301725	*REEP3*	C	T	TG	0.00324
rs12242953	10:70865342	*SRGN*	G	A	T2D	0.001703
rs7901016	10:74637326	*MCU*	T	C	LDL-C	0.013638
rs10824026	10:75421208	*SYNPO2L*	G	A	AF	0.008192
rs12571751	10:80942631	*ZMIZ1*	A	G	T2D	0.012842
rs17680741	10:82251514	*TSPAN14*	C	T	CAD	0.017286
rs1412444	10:91002927	*LIPA*	T	C	CAD	0.019499
rs7087591	10:94473629	*Y_RNA*	G	A	T2D	0.018542
rs2068888	10:94839642	*CYP26A1*	G	A	TG;HDL-C	0.025356
rs9663362	10:95895177	*PLCE1*	C	G	BP	0.002532
rs7917772	10:104487443	*SFXN2*	G	A	WHR	0.001358
rs11191416	10:104604916	*PFN1P11*	G	T	CAD	0.000639
rs4409766	10:104616663	*BORCS7*	T	C	BP	0.02093
rs12415501	10:105324774	*NEURL1*	T	C	AF	0.01344
rs2295786	10:105616482	*SH3PXD2A*	A	T	stroke	0.028779
rs4918072	10:105693644	*STN 1.00*	A	G	CAD	0.019418
rs1129555	10:113910721	*GPAM*	A	G	LDL-C;TC	0.006793
rs2297991	10:113913222	*GPAM*	T	C	HDL-C;TC	0.002211
rs7903146	10:114758349	*TCF7L2*	T	C	T2D	0.031386
rs11196288	10:115057443	*RNU7-165P*	G	A	stroke	0.001458
rs2782980	10:115781527	*ADRB1*	T	C	BP	0.006009
rs10886471	10:121149403	*GRK5*	T	C	T2D	0.002742
rs4752700	10:124237612	*HTRA1*	G	A	CAD	0.01175
rs2334499	11:1696849	*FAM99B*	T	C	T2D	0.016186
rs7107784	11:2215089	*MIR4686*	G	A	T2D	0.012509
rs117601636	11:2642037	*KCNQ1*	A	G	T2D	0.031314
rs2237892	11:2839751	*KCNQ1*	C	T	BMI	0.00276
rs2237896	11:2858440	*KCNQ1*	G	A	T2D	0.019585
rs11601507	11:5701074	*TRIM5*	C	A	CAD	0.004847
rs10160804	11:8600240	*STK33*	A	C	BMI	0.01546
rs93138	11:9759713	*SWAP70*	G	T	CAD	0.024666
rs173396	11:9759918	*SWAP70*	A	G	CAD	0.017575
rs3993105	11:13303071	*ARNTL*	T	C	CAD	0.021612
rs4757391	11:16302939	*SOX6*	T	C	BP	0.000164
rs5215	11:17408630	*KCNJ11*	C	T	T2D	0.01002
rs11030104	11:27684517	*BDNF*	A	G	BMI	0.024809

© 2024 Diao T et al. *JAMA Network Open.*

rs7116641	11:43696917	*RP11-472I20.4*	G	T	CAD	0.006531
rs326214	11:47298360	*MADD*	A	G	HDL-C	0.019189
rs11604680	11:47457539	*RAPSN*	G	A	BMI	0.017627
rs751984	11:61278246	*LRRC10B*	T	C	BP	0.015944
rs174546	11:61569830	*FADS1*	C	T	HDL-C;TG;TC	0.003539
rs174547	11:61570783	*FADS1*	T	C	HDL-C;TC;TG	0.004305
rs12801636	11:65391317	*PCNX3*	G	A	CAD;HDL-C	0.03023
rs1552224	11:72433098	*ARAP1*	A	C	T2D	0.020646
rs590121	11:75274150	*SERPINH1*	T	G	CAD	0.004237
rs499974	11:75455021	*RN7SL786P*	A	C	HDL-C	0.00026
rs10830963	11:92708710	*MTNR1B*	G	C	T2D	0.004251
rs633185	11:100593538	*ARHGAP42*	C	G	BP	0.022775
rs7947761	11:100624599	*ARHGAP42*	G	A	CAD	0.028556
rs660599	11:102729757	*MMP12*	G	A	stroke	0.006967
rs2128739	11:103673277	*RP11-563P16.1*	A	C	CAD	0.009494
rs180327	11:116623659	*BUD13*	T	C	HDL-C;TG	0.002885
rs964184	11:116648917	*ZPR1*	C	G	CAD	0.000924
rs2075291	11:116661392	*APOA5*	A	C	TG;HDL-C	0.051598
rs651821	11:116662579	*APOA5*	C	T	TC;TG;HDL-C	0.025338
rs12718465	11:116707736	*APOA1*	C	T	HDL-C	0.019784
rs17122278	11:118449370	*ARCN1*	A	G	TC	8.20E-05
rs17135399	11:126218541	*DCPS*	G	A	LDL-C	0.02124
rs11222084	11:130273230	*ADAMTS8*	A	T	BP	0.002906
rs4766228	12:4363420	*CCND2-AS1*	A	G	T2D	0.019459
rs11838267	12:7175872	*C1S*	T	C	CAD	0.007294
rs4883263	12:7649484	*CD163*	T	C	HDL-C	0.008395
rs4883201	12:9082581	*PHC1*	A	G	TC	0.001736
rs7980458	12:20162571	*RP11-405A12.2*	G	T	BP	0.014841
rs3861086	12:20218869	*RP11-664H17.1*	C	T	CAD	0.010212
rs7304841	12:20577593	*PDE3A*	A	C	stroke	0.026809
rs1027087	12:26470850	*RP11-283G6.4*	A	T	WHR	0.000408
rs10842992	12:27963839	*RN7SKP15*	T	C	T2D	0.013133
rs80234489	12:31441179	*FAM60A*	C	A	T2D	0.007732
rs897057	12:50266279	*FAIM2*	C	T	BMI	0.013906
rs7306523	12:53393964	*EIF4B*	A	G	LDL-C;TC	0.002059
rs3809128	12:56709919	*CNPY2*	C	T	WC	0.044258
rs2261181	12:66212318	*RPSAP52*	T	C	T2D	5.90E-05
rs7955901	12:71433293	*CTD-2021H9.2*	C	T	T2D	0.008204

© 2024 Diao T et al. *JAMA Network Open.*

rs17249754	12:90060586	*ATP2B1*	G	A	BP	0.018378
rs7136259	12:90081188	*ATP2B1*	C	T	CAD	0.02906
rs7306455	12:95355541	*NDUFA12*	G	A	CAD	0.00481
rs4923678	12:95368163	*NDUFA12*	A	G	CAD	0.011429
rs34008534	12:95381088	*NDUFA12*	A	G	CAD	0.001313
rs7965082	12:100800193	*SLC17A8*	C	T	LDL-C;TC	0.013197
rs2075260	12:109696838	*ACACB*	A	G	TG	0.003984
rs9593	12:109994870	*MMAB*	T	A	HDL-C	0.001872
rs7134594	12:110000193	*MMAB*	T	C	HDL-C	0.002283
rs12229654	12:111414461	*LINC01405*	T	G	BMI	0.070166
rs6490029	12:111698457	*CUX2*	A	G	AF	0.060473
rs671	12:112241766	*ALDH2*	G	A	HDL-C	0.059611
rs11066280	12:112817783	*HECTD4*	T	A	CAD	0.0435
rs10507248	12:114797093	*TBX5*	T	G	AF	0.016668
rs35444	12:115552437	*RP11-25E2.1*	A	G	BP;stroke	0.059406
rs11067763	12:116198341	*RP11-110L15.1*	A	G	BP	0.007334
rs11830157	12:118265441	*KSR2*	G	T	CAD	0.000413
rs1169288	12:121416650	*HNF1A*	C	A	LDL-C;TC	0.009642
rs55783344	12:121432299	*HNF1A*	T	C	T2D	0.0061
rs2258287	12:121454313	*NA*	C	A	CAD	0.004674
rs3213545	12:121471337	*OASL*	G	A	CAD	0.000326
rs4275659	12:123447928	*ABCB9*	T	C	T2D	0.004216
rs10773003	12:123775127	*SBNO1*	A	G	HDL-C	0.005781
rs838880	12:125261593	*SCARB1*	C	T	HDL-C	0.004594
rs11057830	12:125307053	*SCARB1*	A	G	CAD	0.002602
rs12581963	12:125317125	*SCARB1*	G	A	CAD	0.01256
rs9552911	13:23864657	*SGCG*	G	A	T2D	0.004508
rs9512699	13:28029896	*MTIF3*	G	A	BMI	0.00127
rs9319428	13:28973621	*FLT1*	A	G	CAD	0.000839
rs9534262	13:32936646	*BRCA2*	T	C	LDL-C	0.005058
rs9591012	13:33058333	*N4BP2L2*	G	A	CAD	0.000126
rs1467605	13:47211861	*LRCH1*	A	C	stroke	0.006693
rs9568867	13:54107352	*RP11-384G23.1*	A	G	BMI	0.013855
rs1359790	13:80717156	*RP11-470M1.2*	G	A	T2D	0.00242
rs7989336	13:97017548	*HS6ST3*	A	G	BMI	0.002368
rs11838776	13:111040681	*COL4A2*	A	G	CAD	0.025916
rs1317507	13:113631780	*NA*	A	C	CAD	0.009904
rs2415317	14:36609678	*LINC00609*	G	A	stroke	0.000631
rs1982963	14:52509101	*NID2*	A	G	WHR	0.010761
rs2145598	14:58794001	*ARID4A*	G	A	CAD	0.001432
rs1152591	14:64680848	*SYNE2*	G	A	AF	0.003268
rs11624704	14:78786077	*NRXN3*	C	A	BMI	0.016364

© 2024 Diao T et al. *JAMA Network Open.*

rs10139550	14:100145710	*HHIPL1*	G	C	CAD	0.016338
rs7403531	15:38822905	*NA*	T	C	T2D	0.016541
rs67839313	15:40619724	*C15orf52*	T	C	T2D	0.004646
rs4471613	15:58551694	*intergenic (near ALDH1A2, AQP9, LIPC)*	G	A	stroke	0.002689
rs2043085	15:58680954	*ALDH1A2*	T	C	HDL-C;TG;TC	0.004497
rs1532085	15:58683366	*NA*	A	G	TC;TG;HDL-C	0.00735
rs1077834	15:58723479	*LIPC*	C	T	HDL-C;TC	0.014756
rs1800588	15:58723675	*LIPC*	T	C	TG;TC;HDL-C	0.001532
rs1436953	15:62414014	*NPM1P47*	C	T	T2D	0.000256
rs6494488	15:65024204	*RBPMS2*	A	G	CAD	0.002734
rs56062135	15:67455630	*SMAD3*	C	T	CAD	0.083013
rs4776970	15:68080886	*MAP2K5*	A	T	BMI	0.008888
rs7164883	15:73652174	*HCN4*	A	G	AF	0.019479
rs1378942	15:75077367	*CSK*	C	A	BP	0.018302
rs7178572	15:77747190	*HMG20A*	G	A	T2D	0.010484
rs12438008	15:79084680	*ADAMTS7*	A	G	CAD	0.006657
rs4468572	15:79124475	*MORF4L1*	C	T	CAD	0.008664
rs11634397	15:80432222	*ZFAND6*	G	A	T2D	0.002141
rs8030379	15:84590910	*ADAMTSL3*	A	G	WC	0.034258
rs8042271	15:89574218	*RP11-326A19.2*	G	A	CAD	0.004003
rs2028299	15:90374257	*AP3S2*	C	A	T2D	0.010639
rs4932370	15:91404705	*RN7SL363P*	A	G	stroke	0.011905
rs17514846	15:91416550	*FURIN*	A	C	CAD	0.041377
rs1029420	15:91441086	*MAN2A2*	C	T	BP	0.008398
rs79548680	15:91505779	*RCCD1*	C	G	T2D	0.003824
rs17581137	15:96146414	*RP11-61O11.1*	C	A	CAD	0.00358
rs2531995	16:4013467	*ADCY9*	T	C	BMI	0.008613
rs12597579	16:20257867	*GP2*	C	T	BMI	0.01756
rs4788102	16:28873398	*SH2B1*	A	G	BMI	0.02254
rs1421085	16:53800954	*FTO*	C	T	T2D	0.000831
rs1558902	16:53803574	*FTO*	A	T	BMI	0.033351
rs247616	16:56989590	*AC012181.1*	C	T	HDL-C;TC	0.002768
rs7499892	16:57006590	*CETP*	T	C	TG;HDL-C;LDL-C	0.011652
rs2303790	16:57017292	*CETP*	A	G	HDL-C	0.068929
rs2292318	16:67985706	*SLC12A4*	C	T	HDL-C	0.001683
rs3785100	16:67997920	*SLC12A4*	C	T	HDL-C	0.000792

© 2024 Diao T et al. *JAMA Network Open.*

rs17358402	16:71967927	*PKD1L3*	C	T	TC;LDL-C	0.008476
rs7185272	16:72013797	*PKD1L3*	C	G	LDL-C;TC	0.002115
rs12927205	16:72025077	*PKD1L3*	A	G	LDL-C;TC	0.00263
rs2000999	16:72108093	*HPR*	A	G	TC;LDL-C	0.01412
rs7193343	16:73029160	*ZFHX3*	T	C	stroke	0.015964
rs2106261	16:73051620	*ZFHX3*	T	C	AF	0.008417
rs12932445	16:73069888	*ZFHX3*	C	T	stroke	0.014001
rs7202877	16:75247245	*CTRB1*	T	G	T2D	0.024281
rs7206541	16:75436713	*CFDP1*	T	A	CAD	0.031182
rs1424233	16:79682751	*AC009159.1*	C	T	BMI	0.000984
rs2925979	16:81534790	*CMIP*	T	C	HDL-C	0.014501
rs7199941	16:81906423	*PLCG2*	G	A	CAD	0.001089
rs7500448	16:83045790	*CDH13*	A	G	CAD	0.020955
rs12445022	16:87575332	*RP11-482M8.1*	A	G	stroke	0.026428
rs216172	17:2126504	*SMG6*	C	G	CAD	0.01141
rs391300	17:2216258	*SRR*	T	C	T2D	0.008666
rs13342232	17:6945940	*SLC16A11*	G	A	T2D	0.021048
rs12936587	17:17543722	*RP11-524F11.3*	A	G	CAD	0.009267
rs13723	17:27941886	*CORO6*	G	A	CAD	0.016961
rs76954792	17:30033514	*RP11-805L22.1*	T	C	CAD	0.001032
rs7208487	17:37543449	*FBXL20*	G	T	HDL-C	0.00324
rs11869286	17:37813856	*STARD3*	G	C	HDL-C	0.006136
rs2074158	17:40257163	*DHX58*	C	T	CAD	0.017848
rs12946454	17:43208121	*PLCD3*	A	T	BP	0.002922
rs17608766	17:45013271	*GOSR2*	C	T	CAD	0.006628
rs9299	17:46669430	*HOXB5*	T	C	BMI	0.015006
rs7405452	17:46674670	*HOXB6*	C	T	BP	0.024518
rs46522	17:46988597	*UBE2Z*	T	C	CAD	0.019302
rs7213603	17:59003189	*BCAS3*	C	T	CAD	0.004181
rs7225581	17:59017813	*BCAS3*	A	T	CAD	0.008471
rs2240736	17:59485393	*TBX2*	T	C	BP	0.030384
rs1867624	17:62387091	*RPL31P57*	C	T	CAD	0.002388
rs9892152	17:62401965	*RPL31P57*	C	T	CAD	0.013103
rs4148008	17:66875294	*ABCA8*	G	C	HDL-C	0.004386
rs12453914	17:67138878	*ABCA6*	A	C	LDL-C;TC	0.000304
rs11077501	17:68526061	*RP11-1058G23.1*	C	T	WHR	0.004487
rs4129767	17:76403984	*PGS1*	A	G	HDL-C	0.001616
rs7503807	17:78591111	*RPTOR*	A	C	BMI	0.017738
rs8090011	18:7068462	*NA*	G	C	T2D	0.003762
rs1805081	18:21140432	*NPC1*	T	C	BMI	0.002528
rs2000813	18:47093864	*LIPG*	C	T	HDL-C	0.005343

© 2024 Diao T et al. *JAMA Network Open.*

rs12970066	18:47107152	*LIPG*	C	G	HDL-C	0.006678
rs4939883	18:47167214	*SMUG1P1*	C	T	HDL-C;TC	0.001743
rs2156552	18:47181668	*SMUG1P1*	A	T	HDL-C	0.001575
rs11660468	18:47209143	*SMUG1P1*	T	C	CAD	0.002895
rs35337492	18:47210824	*SMUG1P1*	A	G	CAD	0.021699
rs663129	18:57838401	*RNU4-17P*	A	G	CAD	0.033502
rs740406	19:2232221	*NA*	G	A	BP	0.024213
rs2229383	19:10794630	*ILF3-SLC44A2*	T	G	stroke	0.015959
rs7258189	19:11169947	*SMARCA4*	T	C	stroke	0.012802
rs56289821	19:11188247	*LDLR*	A	G	CAD	0.018865
rs7258950	19:11250139	*SPC24*	G	A	LDL-C;TC	0.005481
rs11557092	19:11257018	*SPC24*	C	T	TC;LDL-C	0.019002
rs737337	19:11347493	*DOCK6*	T	C	LDL-C;HDL-C;TC	0.013695
rs73015714	19:17855763	*FCHO1*	G	C	CAD	0.043675
rs58542926	19:19379549	*TM6SF2*	C	T	TC;TG;LDL-C	0.002019
rs10401969	19:19407718	*SUGP1*	T	C	T2D	0.015394
rs16967013	19:32859514	*ZNF507*	G	C	CAD	0.012335
rs29941	19:34309532	*KCTD15*	G	A	BMI	0.014278
rs7258445	19:41855515	*TGFB1*	G	A	CAD	0.013393
rs769449	19:45410002	*APOE*	A	G	TG;HDL-C;LDL-C;TC	0.01558
rs72654473	19:45414399	*APOC1*	C	A	TC;TG	0.0449
rs439401	19:45414451	*APOC1*	C	T	HDL-C;TC;TG	0.000925
rs4420638	19:45422946	*APOC1*	G	A	CAD	0.011064
rs8108269	19:46158513	*RN7SL836P*	G	T	T2D	0.005058
rs2302593	19:46196634	*GIPR*	C	G	CAD	0.010062
rs3810291	19:47569003	*ZC3H4*	A	G	BMI	0.007667
rs17695224	19:52324216	*FPR3*	A	G	HDL-C	0.005723
rs6038557	20:6594349	*RP5-971N18.3*	A	G	WC	0.001235
rs1887320	20:10965998	*RP11-103J8.1*	A	G	BP	0.025805
rs2328223	20:17845921	*RIN2*	C	A	LDL-C	0.00717
rs867186	20:33764554	*PROCR*	A	G	CAD	0.004895
rs4911495	20:33971978	*UQCC1*	A	C	WHR	0.005941
rs6065311	20:39724338	*TOP 1.00*	C	T	LDL-C	0.009579
rs6093446	20:39780932	*PLCG1*	G	A	CAD	0.001469
rs4812829	20:42989267	*HNF4A*	A	G	T2D	0.003827
rs3827066	20:44586023	*ZNF335*	T	C	CAD	0.0148

© 2024 Diao T et al. *JAMA Network Open.*

rs16990971	20:44601293	*ZNF335*	A	G	TG	0.002217
rs13041126	20:51092996	*LINC01524*	C	T	BMI	0.000323
rs2057291	20:57472043	*GNAS*	G	A	CAD;WC	0.022533
rs181359	22:21928641	*UBE2L3*	A	G	HDL-C	0.00503
rs181360	22:21928916	*UBE2L3*	T	G	HDL-C	0.008669
rs16999793	22:24656875	*POM121L9P*	G	C	CAD	0.021758
rs4821382	22:35638115	*SCUBE1*	G	C	WHR	0.012566
rs5996074	22:42236337	*SREBF2*	G	A	BMI	0.006793
rs738409	22:44324727	*PNPLA3*	C	G	TG	0.001979
rs1800234	22:46615880	*PPARA*	T	C	TG	0.009847

Abbreviations: PRS, polygenic risk score; CAD, coronary artery disease; HDL-C, high density lipoprotein cholesterol; LDL-C, low density lipoprotein cholesterol; TC, total cholesterol; TG, triglycerides; BMI, body mass index; WHR, waist to hip ratio; BP, blood pressure; T2D, type 2 diabetes; AF, atrial fibrillation.

© 2024 Diao T et al. *JAMA Network Open.*

eFigure 2. Distributions of CHD PRS and Stroke PRS

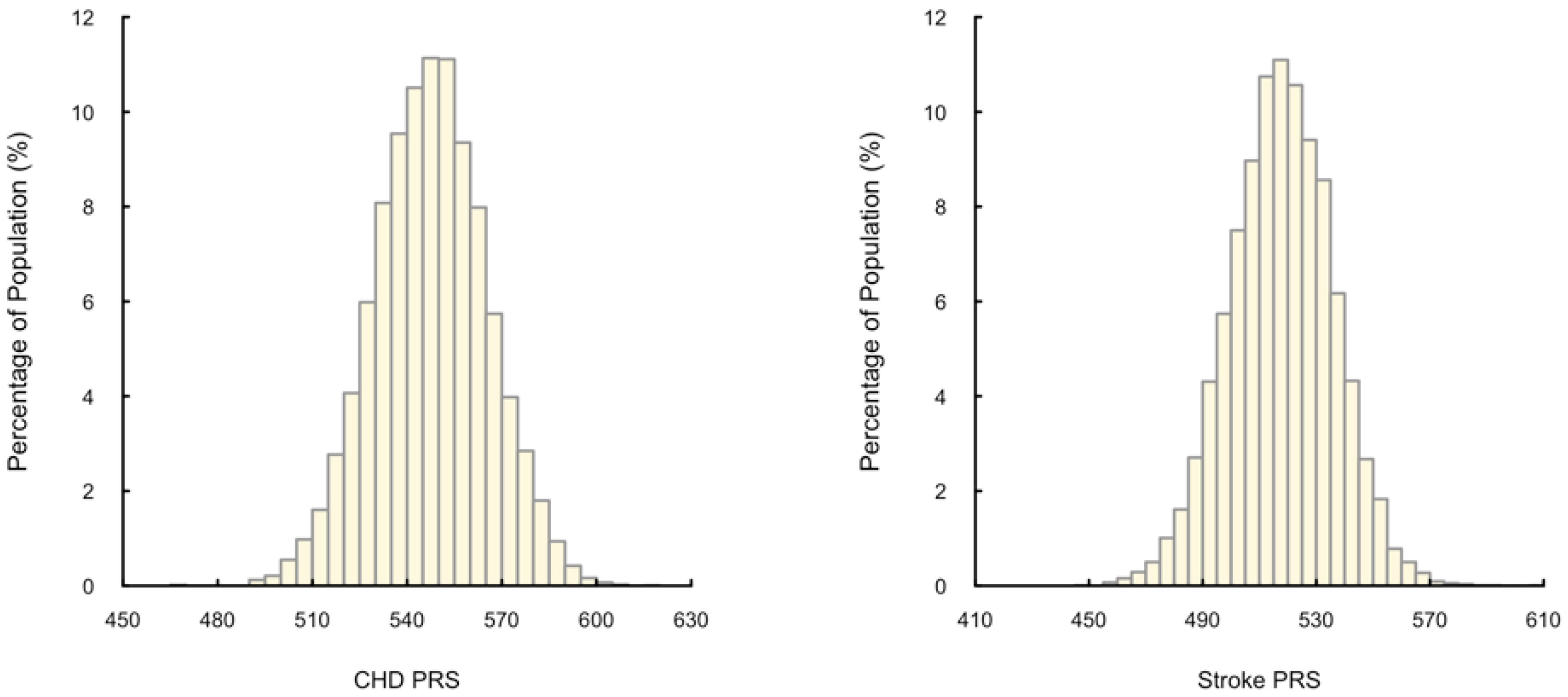

Abbreviations: CHD, coronary heart disease; PRS, polygenic risk score.

© 2024 Diao T et al. *JAMA Network Open.*

eTable 3. Associations of CHD PRS and Stroke PRS With Risk of Incident CHD and Stroke

PRS	CHD		Stroke	
	**Cases/person-years**	**HR (95% CI)** [b]	**Cases/person-years**	**HR (95% CI)** [b]
Low (quintile 1)	464/12 985	1.00 (Reference)	94/13 940	1.00 (Reference)
Intermediate (quintile 2–4)	1491/38 623	1.08 (0.98, 1.20)	333/41 498	1.23 (0.98, 1.54)
High (quintile 5)	576/12 548	1.30 (1.15, 1.46)	118/13 804	1.31 (1.00, 1.72)
Continuous PRS [a]		1.11 (1.07, 1.16)		1.14 (1.05, 1.24)

Abbreviations: PRS, polygenic risk score; CHD, coronary heart disease; HR, hazard ratio; CI, confidence interval.

[a] The z-standardized PRS.

[b] The Cox regression models were adjusted for age and sex.

© 2024 Diao T et al. *JAMA Network Open.*

eTable 4. Associations of Changes in Sleep Patterns With Risk of Incident CVD According to Age and Sex

Subgroups	Sleep pattern change			
	**Persistent unfavorable**	**Favorable-unfavorable**	**Unfavorable-favorable**	**Persistent favorable**
CVD				
Age < 65 years				
Cases/person-years	409/10 480	223/7132	225/6964	313/10 479
HR (95% CI) [a]	1.00 (Reference)	0.83 (0.71, 0.98)	0.88 (0.74, 1.03)	0.83 (0.71, 0.97)
Age ≥ 65 years				
Cases/person-years	1108/15 674	496/8207	400/6860	495/9363
HR (95% CI) [a]	1.00 (Reference)	0.87 (0.78, 0.96)	0.83 (0.74, 0.93)	0.78 (0.70, 0.87)
Male				
Cases/person-years	788/12 030	331/6141	275/5433	352/7165
HR (95% CI) [a]	1.00 (Reference)	0.85 (0.75, 0.96)	0.79 (0.69, 0.91)	0.79 (0.70, 0.90)
Female				
Cases/person-years	729/14 124	388/9198	350/8391	456/12 678
HR (95% CI) [a]	1.00 (Reference)	0.86 (0.76, 0.98)	0.89 (0.79, 1.02)	0.81 (0.71, 0.91)
CHD				
Age < 65 years				
Cases/person-years	339/10 651	193/7217	198/7029	277/10 562
HR (95% CI) [a]	1.00 (Reference)	0.87 (0.73, 1.04)	0.93 (0.78, 1.11)	0.89 (0.75, 1.04)
Age ≥ 65 years				
Cases/person-years	867/16 185	382/8433	325/7070	405/9566
HR (95% CI) [a]	1.00 (Reference)	0.85 (0.75, 0.96)	0.86 (0.76, 0.98)	0.82 (0.72, 0.92)
Male				
Cases/person-years	604/12 389	236/6341	216/5577	277/7345
HR (95% CI) [a]	1.00 (Reference)	0.78 (0.67, 0.91)	0.81 (0.69, 0.94)	0.80 (0.69, 0.93)
Female				
Cases/person-years	602/14 446	339/9310	307/8522	405/12 783
HR (95% CI) [a]	1.00 (Reference)	0.92 (0.81, 1.05)	0.95 (0.83, 1.09)	0.87 (0.77, 0.99)
Stroke				
Age < 65 years				
Cases/person-years	70/11 329	30/7578	27/7443	36/11 217
HR (95% CI) [a]	1.00 (Reference)	0.67 (0.44, 1.03)	0.64 (0.41, 1.00)	0.58 (0.38, 0.88)
Age ≥ 65 years				
Cases/person-years	241/17 733	114/9174	75/7609	90/10 450
HR (95% CI) [a]	1.00 (Reference)	0.94 (0.75, 1.18)	0.74 (0.57, 0.96)	0.69 (0.54, 0.89)
Male				
Cases/person-years	184/13 408	95/6724	59/5923	75/7860

© 2024 Diao T et al. *JAMA Network Open.*

HR (95% CI) [a]	1.00 (Reference)	1.09 (0.85, 1.39)	0.76 (0.57, 1.02)	0.78 (0.59, 1.03)
Female				
Cases/person-years	127/15 654	49/10 028	43/9128	51/13 808
HR (95% CI) [a]	1.00 (Reference)	0.63 (0.45, 0.88)	0.65 (0.46, 0.92)	0.53 (0.38, 0.74)

Abbreviations: CVD, cardiovascular diseases; CHD, coronary heart disease; HR, hazard ratio; CI, confidence interval.

[a] The Cox regression models were adjusted for age, sex, education level, smoking status, drinking status, regular exercise, body mass index, hypertension, diabetes mellitus, hyperlipidemia, and family history of CVD, CHD, or stroke (in the corresponding analysis). The stratified factors were not included in each stratified analysis.

© 2024 Diao T et al. *JAMA Network Open.*

eTable 5. Associations of Changes in Sleep Patterns With Risk of Incident CVD After Excluding Events Occurred Within the First Year of Follow-Up

Changes in sleep pattern	CVD		CHD		Stroke	
	Cases/person-years	HR (95% CI) [a]	Cases/person-years	HR (95% CI) [a]	Cases/person-years	HR (95% CI) [a]
Persistent unfavorable	1222/25 972	1.00 (Reference)	976/26 311	1.00 (Reference)	246/27 867	1.00 (Reference)
Favorable-unfavorable	582/15 252	0.85 (0.77, 0.94)	469/15 413	0.85 (0.76, 0.95)	113/16 163	0.86 (0.69, 1.08)
Unfavorable-favorable	536/13 768	0.89 (0.81, 0.99)	448/13 922	0.92 (0.82, 1.03)	88/14 651	0.77 (0.60, 0.98)
Persistent favorable	663/19 752	0.80 (0.73, 0.88)	552/19 913	0.82 (0.74, 0.91)	111/20 958	0.73 (0.58, 0.92)

Abbreviations: CVD, cardiovascular diseases; CHD, coronary heart disease; HR, hazard ratio; CI, confidence interval.

[a] The Cox regression models were adjusted for age, sex, education level, smoking status, drinking status, regular exercise, body mass index, hypertension, diabetes mellitus, hyperlipidemia, and family history of CVD, CHD, or stroke (in the corresponding analysis).

© 2024 Diao T et al. *JAMA Network Open.*

eTable 6. Associations of Changes in Sleep Patterns With Risk of Incident CVD After Excluding Participants Reporting Very Poor Sleep Quality With Frequent Use of Hypnotics

Changes in sleep pattern	CVD		CHD		Stroke	
	Cases/person-years	HR (95% CI) [a]	Cases/person-years	HR (95% CI) [a]	Cases/person-years	HR (95% CI) [a]
Persistent unfavorable	1454/25 284	1.00 (Reference)	1150/25 945	1.00 (Reference)	304/28 062	1.00 (Reference)
Favorable-unfavorable	700/14 989	0.86 (0.79, 0.94)	556/15 295	0.86 (0.78, 0.95)	144/16 359	0.89 (0.73, 1.08)
Unfavorable-favorable	617/13 675	0.85 (0.78, 0.94)	516/13 949	0.89 (0.80, 0.99)	101/14 887	0.71 (0.57, 0.89)
Persistent favorable	808/19 712	0.81 (0.74, 0.89)	682/19 997	0.86 (0.78, 0.94)	126/21 537	0.67 (0.54, 0.82)

Abbreviations: CVD, cardiovascular diseases; CHD, coronary heart disease; HR, hazard ratio; CI, confidence interval.

[a] The Cox regression models were adjusted for age, sex, education level, smoking status, drinking status, regular exercise, body mass index, hypertension, diabetes mellitus, hyperlipidemia, and family history of CVD, CHD, or stroke (in the corresponding analysis).

© 2024 Diao T et al. *JAMA Network Open.*

eFigure 3. Spearman Correlation Between Individual Sleep Factors

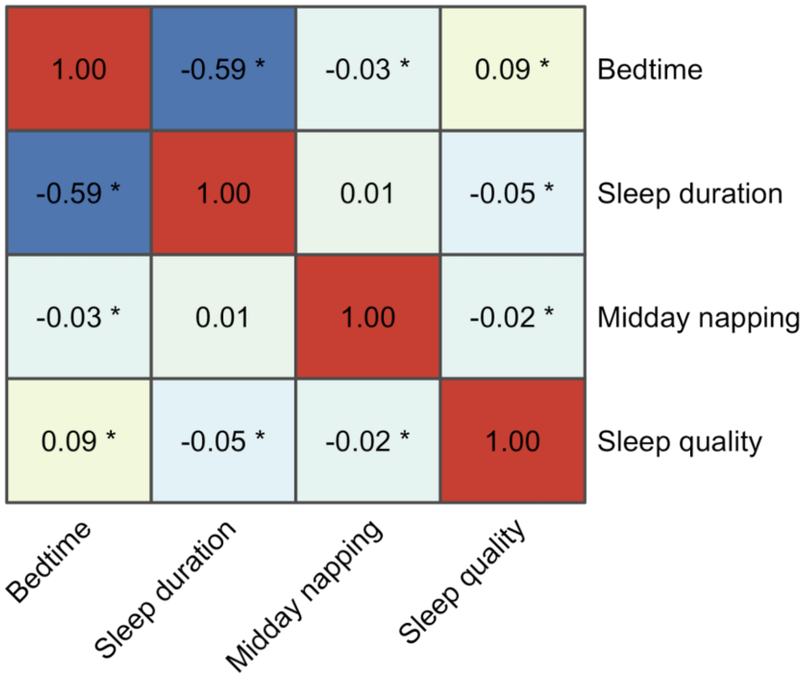

Spearman correlation coefficients between individual sleep factors are presented. Bedtime, sleep duration, and midday napping were continuous variables. Sleep quality was coded as good, fair, and poor. *P <.05

© 2024 Diao T et al. *JAMA Network Open.*

eReferences

1. Prather AA, Epel ES, Cohen BE, Neylan TC, Whooley MA. Gender differences in the prospective associations of self-reported sleep quality with biomarkers of systemic inflammation and coagulation: findings from the Heart and Soul Study. *J Psychiatr Res.* 2013;47(9):1228-1235. doi:10.1016/j.jpsychires.2013.05.004.
2. Wang C, Bangdiwala SI, Rangarajan S, et al. Association of estimated sleep duration and naps with mortality and cardiovascular events: a study of 116 632 people from 21 countries. *Eur Heart J.* 2019;40(20):1620-1629. doi:10.1093/eurheartj/ehy695.
3. Wang C, Hu B, Rangarajan S, et al. Association of bedtime with mortality and major cardiovascular events: an analysis of 112,198 individuals from 21 countries in the PURE study. *Sleep Med.* 2021;80:265-272. doi:10.1016/j.sleep.2021.01.057.
4. Mu X, Yu K, Long P, et al. Leisure-time physical activity and risk of incident cardiovascular disease in Chinese retired adults. *Sci Rep.* 2021;11(1):24202. doi:10.1038/s41598-021-03475-6.

© 2024 Diao T et al. *JAMA Network Open.*
