# Supplementary material for: Changes in Sleep Patterns, Genetic Susceptibility, and Incident Cardiovascular Disease in China
Source: JAMA Netw Open. 2024 Apr 23;7(4):e247974. doi: 10.1001/jamanetworkopen.2024.7974 (PMC11040405; doi:10.1001/jamanetworkopen.2024.7974)
Supplement: Supplement 2. — Data Sharing Statement [file jamanetwopen-e247974-s002.pdf]

Data Sharing Statement

Diao. Changes in Sleep Patterns, Genetic Susceptibility, and Incident Cardiovascular Disease in China. *JAMA Netw Open*. Published April 23, 2024.
doi:10.1001/jamanetworkopen.2024.7974

Data
Data available: No
